# Effects of adiposity, insulin resistance, and inflammation on cardiometabolic multimorbidity: insights from interaction analyses and metabolic phenotyping in the China health and retirement longitudinal study 2011–2018

**DOI:** 10.1186/s12933-026-03234-9

**Published:** 2026-06-03

**Authors:** Lili You, Wenbo Zhao, Meiguang Zheng, Xiaosi Hong, Meng Ren, Wenpeng Li

**Affiliations:** 1 The Department of Endocrinology, Sun Yat-sen Memorial Hospital, Sun Yat-sen University, Guangzhou, China; 2 The Department of Neurosurgery, Second Hospital of Shanxi Medical University, Taiyuan, 030001 Shanxi China; 3 The Department of Neurosurgery, Sun Yat-sen Memorial Hospital, Sun Yat-sen University, Guangzhou, China

**Keywords:** Cardiometabolic multimorbidity (CMM), Adiposity, Insulin resistance, Metabolic phenotypes, K-means clustering

## Abstract

**Background:**

Cardiometabolic multimorbidity (CMM) burdens aging populations. Obesity drives CMM via insulin resistance and inflammation, but their nonlinear and combined effects remain unclear. We elucidated how these factors contribute to CMM incidence.

**Methods:**

From CHARLS 2011 to 2018, 6,510 participants were enrolled. CMM was defined as ≥ 2 of diabetes, heart disease, and stroke. Cox regression, Kaplan-Meier, and Fine-Gray models were used. Restricted cubic splines (RCS) evaluated nonlinear relationships. Multiplicative and additive interactions were assessed, and mediation analysis with 1,000 bootstraps estimated indirect effects. K-means clustering based on eight standardized variables, including age, body mass index (BMI), waist circumference (WC), triglyceride-glucose (TyG), high-sensitivity C-reactive protein (hs-CRP), systolic blood pressure (SBP), high-density lipoprotein cholesterol (HDL-C), and fasting plasma glucose (FPG), identified metabolic phenotypes carried high CMM risk.

**Results:**

Over 7 years, 212 (3.26%) developed CMM. RCS revealed a J-shaped association between WC and CMM. Optimal cut-offs were 60 years for age, 25.6 kg/m² for BMI, 90.6 cm for WC, 8.7 for the TyG index, 154.3 mg/dL for LDL-C, and 0.86 mg/L for hs-CRP. All six parameters independently predicted CMM. No significant additive interactions were found, but dual elevation markedly increased risk. The TyG index mediated 14.6% of the BMI effect and 10.0% of the WC effect on CMM. Clustering identified insulin-resistant and obese-insulin-resistant phenotypes.

**Conclusion:**

Optimal cut-offs offer practical screening tools. Dual elevation markedly increases CMM risk and insulin resistance mediates adiposity effects. Clustering identified insulin-resistant and obese-insulin-resistant phenotypes, supporting phenotype-based prevention.

**Graphical Abstract:**

**Figure:**
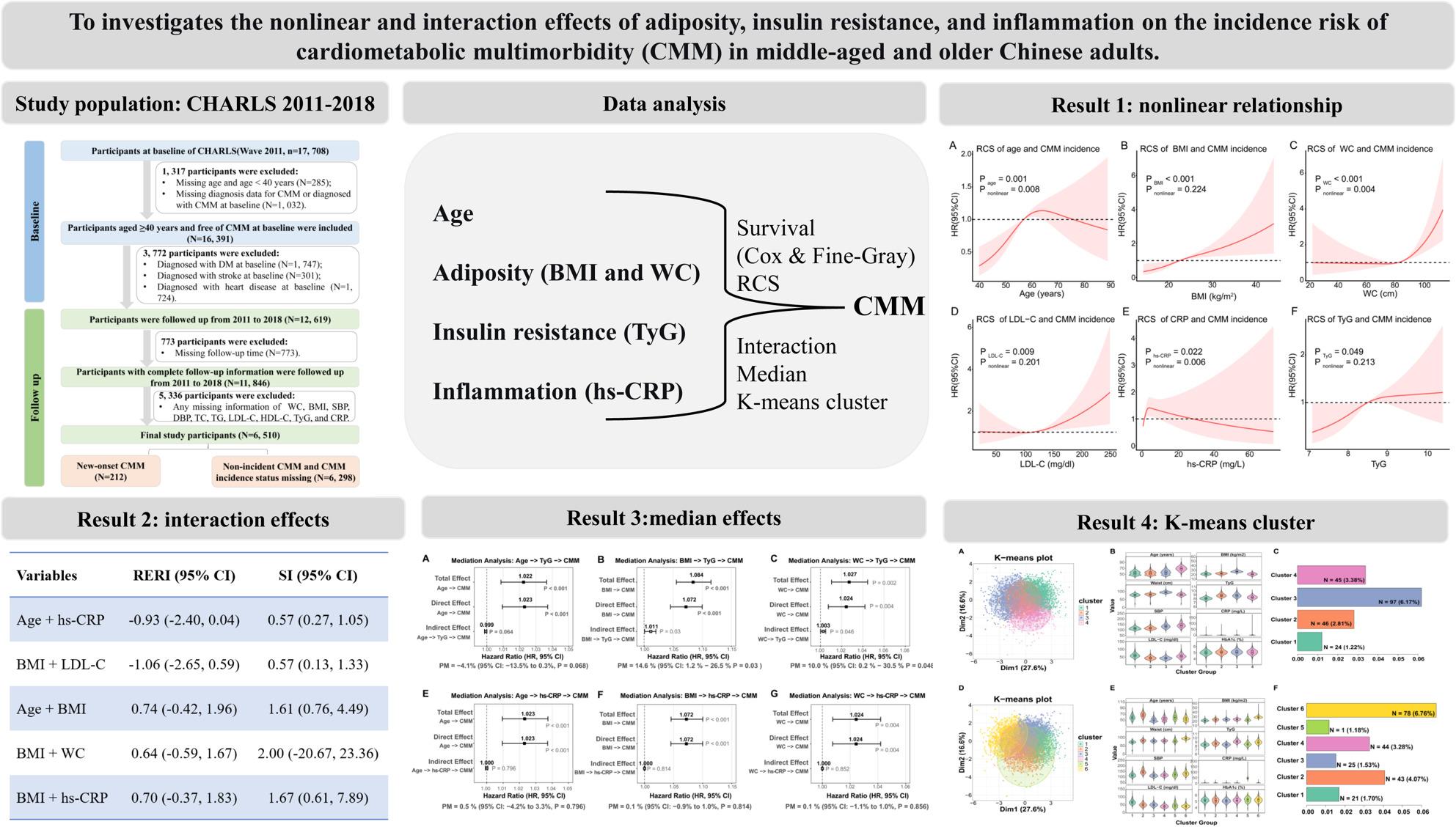

**Supplementary Information:**

The online version contains supplementary material available at 10.1186/s12933-026-03234-9.

## Research insights

**What is currently known about this topic?**

CMM is a growing health burden in aging populations, driven by obesity, insulin resistance, and chronic inflammation. BMI, WC, TyG, and hs-CRP have been identified as independent predictors of CMM risk.

**What is the key research question?**

This study investigates whether adiposity, insulin resistance, and inflammation have nonlinear and combined effects on incident CMM, and further explores their curvilinear relationships, multiplicative and additive interactions, mediating pathways, and data-driven metabolic phenotypes to inform targeted prevention strategies.

**What is new?**

This study identifies nonlinear associations between metabolic indicators and CMM, and demonstrates that dual risk factor elevation amplifies CMM risk, quantifies TyG mediation in the adiposity-CMM pathway. K-means clustering identifies insulin-resistant and obese-insulin-resistant subtypes with the highest CMM risk.

**What are the implications of this study for clinical practice?**

Optimal cut-offs (Age > 60 years, BMI > 25.6 kg/m², WC > 90.6 cm, TyG > 8.7, LDL-C > 154.3 mg/dL, and hs-CRP > 0.86 mg/L) offer practical screening tools. Dual elevation amplifies risk, favoring combined screening. Insulin resistance mediates adiposity effects and represents a therapeutic target. Clustering identifies insulin-resistant and obese-insulin-resistant phenotypes as highest-risk subgroups, enabling phenotype-based prevention.

## Introduction

Global population aging has made multimorbidity an increasingly important healthcare challenge [1]. Cardiometabolic multimorbidity (CMM) is characterized by the presence of two or more conditions among diabetes mellitus, heart disease and stroke [2]. CMM is associated with disability, cognitive impairment [3–5], increased mortality, and reduced life expectancy [6]. Early detection and intervention can support healthy aging and lower healthcare costs. Obesity is recognized as a key driver of CMM [7–9], with underlying mechanisms involving adipose accumulation, insulin resistance and inflammation [10]. These factors interact to promote atherosclerosis, endothelial dysfunction, and target organ damage, ultimately leading to multiple cardiometabolic diseases [11]. Although body mass index (BMI) has traditionally been used to assess obesity-related risk [7], abdominal fat accumulation is a stronger predictor of cardiometabolic complications than BMI [9]. A UK Biobank study of 374,274 adults with 13.7 years of follow-up identified insulin resistance as a predictor of CMM risk [12]. Another UK Biobank study of over 270,000 participants found that chronic low-grade inflammation markers, including high-sensitivity C-reactive protein (hs-CRP) and platelet count, were associated with an increased risk of CMM [13]. A China cohort study of 4,483 participants showed that the triglyceride-glucose (TyG) index and hs-CRP jointly increase CMM risk, with the greatest risk when both are elevated [14]. Collectively, these findings indicate that obesity, insulin resistance, and chronic low-grade inflammation are interrelated drivers of CMM, supporting the integrated assessment of these metabolic parameters to identify high-risk individuals and guide early prevention.

However, the optimal cut-offs, interactions, mediating pathways, and phenotype-based subgroups of these metabolic parameters remain unclear, and their combined roles in CMM have not been examined. To address these gaps, we used data from the China Health and Retirement Longitudinal Study (CHARLS), a nationally representative cohort. We examined the associations of BMI, waist circumference (WC), low-density lipoprotein cholesterol (LDL-C), the TyG index, hs-CRP, and age with CMM, determined optimal cut-offs, evaluated additive interactions and mediating mechanisms, and identified metabolic subtypes via cluster analysis.

## Methods

### Study design and participants

**Fig. 1:**
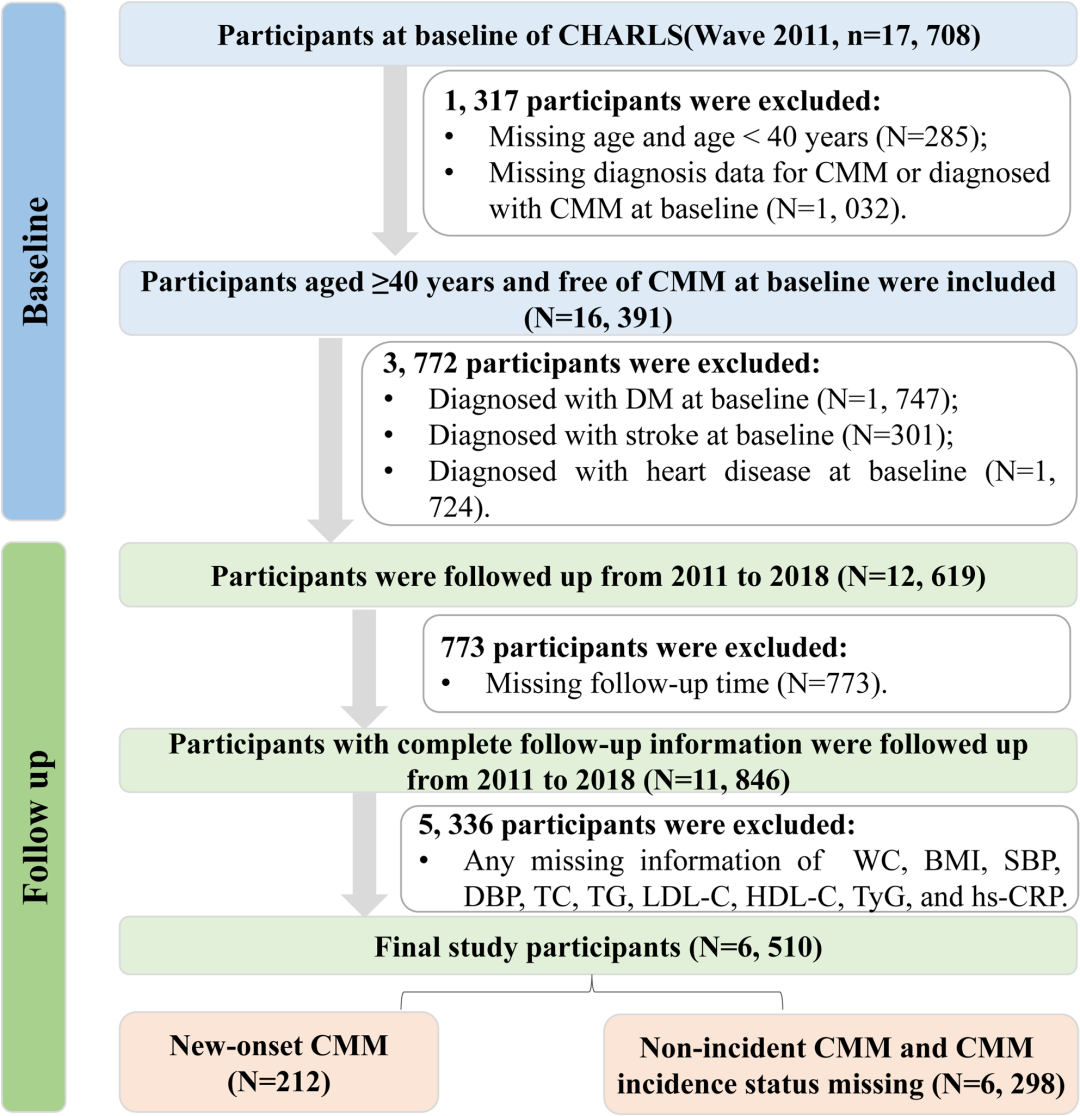
Flowchart of the study population

This study used baseline data from the 2011 wave of the China Health and Retirement Longitudinal Study (CHARLS; http://charls.pku.edu.cn/), with follow-up outcomes ascertained from the 2013, 2015, and 2018 waves. CHARLS is a prospective nationwide cohort study among middle-aged and elderly populations in China. The baseline survey, conducted between June 2011 and March 2012, included a representative sample of 17,708 participants recruited from 450 villages and urban communities across 28 provinces. All participants provided written informed consent, and the study protocol was approved by the Biomedical Ethics Review Committee of Peking University (IRB00001052-11015). Participants were excluded based on the following criteria: (1) missing age or age < 40 years (*N* = 285); (2) missing diagnosis data for CMM or diagnosed with CMM at baseline (*N* = 1,032), leaving 16,391 participants aged ≥ 40 years and free of CMM at baseline; (3) diagnosed with diabetes mellitus, stroke, or heart disease at baseline (*N* = 3,772); (4) missing follow‑up time information (*N* = 773); and (5) any missing information on WC, BMI, SBP, diastolic blood pressure (DBP), total cholesterol (TC), triglycerides (TG), LDL‑C, high-density lipoprotein cholesterol (HDL‑C), TyG, and hs-CRP (*N* = 5,336). A total of 6,510 participants were eligible for analysis (Fig. 1). Compared with excluded participants, included individuals had a lower proportion of males (46.5% vs. 48.7%) and were younger (57.96 vs. 58.81 years). The excluded group showed higher fasting plasma glucose (FPG), glycated hemoglobin (HbA1c) and TG, with standardized mean difference (SMD) of 0.313 to 0.631, consistent with the exclusion of baseline diabetes. The included sample showed no substantial bias relative to the overall cohort, indicating satisfactory representativeness (Table S1).

## Data collection and measurement

Demographic and clinical data were obtained via standardized questionnaires and physical examinations. These included age, sex, height, weight, WC, residence (urban/rural), marital status (married/other), education level (below high school/high school or above), smoking status (yes/no) and drinking status (yes/no), and physician-diagnosed or self-reported conditions. Height was measured using a vertical stadiometer, and weight was measured with participants barefoot and wearing light clothing. WC was taken horizontally at the umbilical level. Blood pressure (BP) was determined as the average of triplicate readings using an Omron HEM-7200 sphygmomanometer. HbA1c and platelet count were measured within 2 h using 2‑mL blood samples. Fasting venous blood was kept at -20 °C and transported under cold-chain conditions. TC, TG, HDL-C, LDL-C, and FPG were quantified using enzymatic colorimetric assays. hs-CRP was measured using an immunoturbidimetric assay on a Hitachi 7180 chemistry analyzer (Hitachi, Tokyo, Japan). The coefficient of variation for all blood biomarkers was below 5%, indicating high reliability [15]. The TyG index was calculated as a measure of insulin resistance, with the formula detailed below [16]: Ln [TG (mg/dL) × FPG (mg/dL) / 2].

## Assessment of obesity and metabolic conditions

According to the criteria of the Working Group on Obesity in China (WGOC), general obesity was defined by BMI. Participants were categorized as obese (BMI ≥ 28.0 kg/m^2^), overweight (BMI 24.0–27.9 kg/m^2^), or normal weight (BMI 18.5–23.9 kg/m^2^) [17]. According to the standards for defining abdominal obesity in China, the WC thresholds for diagnosing abdominal obesity were set at ≥ 90 cm for men and ≥ 85 cm for women [18]. Hypertension was defined as SBP ≥ 140 mmHg, DBP ≥ 90 mmHg, current use of antihypertensive medication, or self-reported history of hypertension [19]. Metabolic syndrome (MetS) was diagnosed when three or more of the following five components were present: (1) abdominal obesity (WC ≥ 90 cm for men and ≥ 80 cm for women); (2) elevated triglycerides (TG ≥ 150 mg/dL); (3) reduced high-density lipoprotein cholesterol (HDL‑C < 40 mg/dL for men and < 50 mg/dL for women); (4) elevated blood pressure (SBP ≥ 130 mm Hg and/or DBP ≥ 85 mm Hg, or use of antihypertensive therapy); and (5) FPG ≥ 100 mg/dL, or use of antidiabetic medications, or self-reported history of diabetes [20]. Pre‑diabetes was defined according to American Diabetes Association (ADA) criteria as the presence of impaired fasting glucose (FPG 100–125 mg/dL) and/or elevated HbA1c (5.7%-6.4%), in the absence of a prior diabetes diagnosis or use of antidiabetic medications [21].

## Assessment of CMM events and time-to-event framework

Incident CMM was defined as the occurrence of at least two of three conditions including diabetes, heart disease, and stroke during follow-up [2]. Diabetes was determined by FPG ≥ 7.0 mmol/L, HbA1c ≥ 6.5%, use of antidiabetic medications, or self-reported physician diagnosis [21]. Heart disease and stroke were ascertained through self-reported physician diagnosis based on standardized questions collected at baseline and follow-up surveys, or via use of relevant cardiovascular medications. After the exclusion of participants with prevalent CMM at baseline, the follow-up period for survival analysis began at the date of the first CHARLS wave (2011). Participants were followed until the first occurrence of any of the following events: (1) new-onset CMM; (2) death; (3) loss to follow-up; or (4) the end of the study period (2018 wave), whichever occurred first [22]. The stepwise selection of the analytical sample is illustrated in Fig.S1. In the competing risks analysis, all‑cause mortality occurring prior to the development of CMM was treated as the competing event, and death was ascertained through proxy reports from family members and verified against official death records where available.

## K-means clustering analysis

K-means clustering was applied to baseline characteristics to identify distinct metabolic phenotypes. Eight indicators including age, BMI, WC, hs-CRP, TyG, SBP, HDL-C, and FPG were standardized as Z-scores. The optimal number of clusters was determined by the elbow method (Fig. S2) and within-cluster sum of squares (WCSS). We examined 4- to 7-cluster solutions, with the 4- and 6-cluster solutions as primary results and the others as sensitivity analyses. The algorithm was implemented using the kmeans() function in R with 50 random initializations and a fixed seed. Cluster stability was evaluated by repeating the clustering procedure over multiple independent runs and calculating the adjusted Rand index (ARI) for all pairwise comparisons across runs. Cluster separation was assessed using the average silhouette width across a range of K values. Each phenotype was subsequently characterized by its unique metabolic profile, and the clinical relevance of the identified clusters was validated through violin plots, radar plots, cumulative incidence curves, and Cox proportional hazards regression models comparing CMM incidence across clusters.

### Statistical analysis

Continuous variables were expressed as mean (SD) for normally distributed data and median (IQR) for non-normally distributed data, with normality assessed using the Shapiro-Wilk test and visual inspection of Q-Q plots prior to selecting parametric or non-parametric tests. Categorical variables were expressed as frequencies and percentages. Group differences were assessed using Student’s t-test, Mann–Whitney U test, chi-squared test, or Fisher’s exact test as appropriate. Relationships among variables were examined using Pearson correlation and displayed as a clustered heatmap. Restricted cubic spline (RCS) based on Cox models was used to assess nonlinear associations between metabolic parameters and CMM incidence, with three knots at the 10th, 50th, and 90th percentiles and the reference set at the median. All models were adjusted for sex, SBP, HbA1c, smoking, drinking, and metabolic parameters (age, WC, LDL-C, hs-CRP, and TyG) not tested as the exposure. Optimal cut-off values were determined using maximally selected rank statistics, and participants were dichotomized into low and high groups based on these cut-offs.

CMM incidence differences between groups were assessed using log-rank tests and Kaplan-Meier survival analyses. Cox proportional hazards regression models were used to estimate hazard ratios (HRs) and 95% confidence intervals (CIs). The proportional hazards (PH) assumption was tested using scaled Schoenfeld residuals; variables violating the assumption were addressed by stratifying Cox models in sensitivity analyses, after which the global PH test was non-significant. Cumulative incidence function (CIF) and Gray’s test were performed, and Fine-Gray competing risks models were used to account for all-cause mortality, with subdistribution hazard ratios (SHRs) reported. To minimize reverse causation, two lag analyses excluded participants with CMM or death within the first 2 years, and both Cox and Fine-Gray models were re-fitted. Model 1 was unadjusted. Model 2 adjusted for sex (male, female), smoking (current, former, never), drinking (current, former, never), SBP (continuous), HbA1c (continuous), metabolic parameters (age, WC, LDL-C, hs-CRP, TyG; all continuous) not tested as the exposure. Multicollinearity was assessed using variance inflation factors (VIF), with all values below 5, indicating no significant collinearity (Table S2).

CMM incidence rates were calculated per 1,000 person-years. Multiplicative and additive interactions between pairs of metabolic parameters were assessed. For multiplicative interaction, a product term of the two exposures (e.g., Age × BMI) was included in Cox proportional hazards models, with P values from Wald tests adjusted for multiple comparisons using the Benjamini-Hochberg FDR correction. Additive interaction was evaluated using relative excess risk due to interaction (RERI), synergy index (SI), and attributable proportion due to interaction (AP). All models were adjusted for the unified core covariate set (sex, age, smoking, drinking, SBP, HbA1c, LDL‑C, hs‑CRP, WC, and TyG), excluding the two variables used to define each interaction. Mediation analysis was performed to quantify the extent to which TyG and hs-CRP mediated the associations of age, BMI, and WC with incident CMM. Natural direct effect (NDE), natural indirect effect (NIE), and total effect (TE) were estimated using a counterfactual framework (CMAverse package) with 95% CIs from 1,000 bootstrap resamples. All mediation models were adjusted for sex, SBP, and the metabolic covariates (age, WC, LDL‑C, hs‑CRP, and TyG) not tested as the exposure or mediator in each respective model.

Standard ROC analysis was additionally conducted to assess the overall discriminatory performance of WC for CMM, with the optimal cut-off determined by the Youden index. The optimal WC cutoff (90.6 cm) was compared with Asian clinical standards (≥ 85 cm for men, ≥ 80 cm for women). Sensitivity, specificity, positive predictive value (PPV), negative predictive value (NPV), Youden index, and positive and negative likelihood ratios were calculated for both cutoffs. Of 17,708 baseline participants, we excluded those with age < 40 years or missing age (*n* = 285), missing CMM data (*n* = 359), prevalent CMM (*n* = 673), baseline diabetes (*n* = 1,747), stroke (*n* = 301), heart disease (*n* = 1,724), or missing follow-up (*n* = 773), leaving 11,846 for analysis. Approximately 19.14% of data were missing (Fig. S3). A missingness matrix suggested data were likely missing at random (MAR). Missing values were addressed using multiple imputation by chained equations (MICE) with five imputed datasets, and results were combined using Rubin’s rules. Cox and Fine-Gray models were re-estimated using the imputed dataset and compared with complete-case analysis to assess robustness. Subgroup analyses were performed by sex, age group, education level, marital status, residence, hypertension, metabolic syndrome, abdominal obesity, pre-diabetes, and K-means clusters. Participants were dichotomized using optimal cut-offs, and Cox models were fitted within each subgroup. All statistical analyses were performed using R software (version 4.5.3). A two-sided *P* < 0.05 was considered statistically significant.

## Results

### Baseline characteristics of participants

A total of 6,510 participants were included, with a mean age of 57.96 years and a female proportion of 53.55%. During a 7-year follow-up, 212 participants (3.26%) developed CMM. Participants who developed CMM were older (60.13 vs. 57.88 years, *P* < 0.001) and more likely to be female (61.32% vs. 53.29%, *P* = 0.025). Participants in the CMM group had a higher prevalence of hypertension, obesity, and abdominal obesity (all *P* < 0.001). In addition, individuals who developed CMM had significantly higher levels of SBP, DBP, BMI, WC, TC, TG, LDL-C, FPG, HbA1c, hs-CRP, and TyG, while HDL-C levels were significantly lower (all *P* < 0.05, Table 1). TyG correlated strongly with TG (*r* ≈ 0.90) and LDL-C with TC (*r* = 0.87); TG, WC, BMI, and hs-CRP were positively intercorrelated (*r* = 0.23–0.59); HDL-C correlated negatively with TG, TyG, WC, and BMI (*r*=-0.48 to -0.23); age showed weak correlations with BMI and WC (Fig. S4).

**Table 1:** Baseline characteristics of participants with and without development of CMM during follow-up

Characteristics	Overall	Non-CMM	CMM	*P* value
	*N* = 6510	*N* = 6298	*N* = 212	
Age, years, mean (SD)	57.96 (9.41)	57.88 (9.44)	60.13 (8.31)	< 0.001
Female, n (%)	3486 (53.55)	3356 (53.29)	130 (61.32)	0.025
Married, n (%)	5501 (84.50)	5321 (84.49)	180 (84.91)	0.945
Residence in Rural, n (%)	4364 (67.04)	4226 (67.10)	138 (65.09)	0.591
Below high school level, n (%)	5911 (90.80)	5722 (90.85)	189 (89.15)	0.502
Smoking, n (%)	2030 (31.26)	1973 (31.41)	57 (26.89)	0.186
Drinking, n (%)	2212 (33.99)	2144 (34.06)	68 (32.08)	0.082
Hypertension, n (%)	2263 (34.85)	2140 (34.07)	123 (58.02)	< 0.001
Obesity, n (%)	1814 (27.88)	1712 (27.20)	102 (48.34)	< 0.001
Abdominal obesity, n (%)	2435 (37.40)	2319 (36.82)	116 (54.72)	< 0.001
SBP, mmHg, mean (SD)	127.61 (20.99)	127.32 (20.84)	136.26 (23.62)	< 0.001
DBP, mmHg, mean (SD)	74.65 (12.19)	74.55 (12.14)	77.59 (13.44)	0.001
BMI, kg/m^2^, mean (SD)	23.21 (3.72)	23.15 (3.70)	24.89 (3.87)	< 0.001
WC, cm, mean (SD)	83.33 (12.03)	83.18 (11.93)	87.97 (13.71)	< 0.001
TC, mg/dl, median(Q1 ~ Q3)	189.43 [166.24–213.40]	189.05 [166.24–213.40]	196.39 [172.04-226.93]	0.001
TG, mg/dl, median(Q1 ~ Q3)	100.01 [71.69-144.26]	99.12 [71.69-143.37]	112.84 [84.96-158.86]	< 0.001
HDL-C, mg/dl, median(Q1 ~ Q3)	50.64 [41.75–61.08]	50.64 [41.75–61.08]	48.33 [39.43–57.41]	0.004
LDL-C, mg/dl, median(Q1 ~ Q3)	114.05 [93.56-136.47]	114.05 [93.56-136.08]	121.59 [102.06–147.10]	< 0.001
FPG, mg/dl, median(Q1 ~ Q3)	99.90 [93.06-107.28]	99.90 [92.88-107.28]	102.87[94.77-111.15]	0.002
HbA1c, %, mean (SD)	5.09 (0.40)	5.09 (0.40)	5.18 (0.40)	0.002
Creatinine, mg/dl, median(Q1 ~ Q3)	99.90 [93.06-107.28]	99.90 [92.88-107.28]	102.87 [94.77-111.15]	0.002
CRP, mg/L, median(Q1 ~ Q3)	0.94 (0.52–1.96)	0.92 (0.51–1.94)	1.30 (0.68–2.82)	< 0.001
Uric acid, mg/dl, median(Q1 ~ Q3)	4.25 [3.55–5.09]	4.25 [3.55–5.09]	4.38 [3.55–5.23]	0.174
Hemoglobin, g/dl, mean (SD)	14.32 (2.22)	14.32 (2.22)	14.34 (2.18)	0.917
TyG, mean (SD)	8.54 (0.55)	8.54 (0.55)	8.70 (0.52)	< 0.001

Data are presented as mean (SD), median (IQR), or n (%) as appropriate. CMM cardiometabolic multimorbidity, SBP systolic blood pressure, DBP diastolic blood pressure, BMI body mass index, WC waist circumference, TC total cholesterol, TG triglycerides, HDL-C high-density lipoprotein cholesterol, LDL-C low-density lipoprotein cholesterol, FPG fasting plasma glucose, HbA1c glycated hemoglobin, CRP C-reactive protein, TyG, triglyceride-glucose.

**Table 2:** Associations of baseline metabolic parameters with risk of incident CMM

Variables	Total *N*	Cases *N* (%)	Model 1		Model 2	
			HR (95%CI)	*P* values	HR (95%CI)	*P*
*Age*						
≤ 60	4169	114 (2.73)	1.00	–	1.00	–
> 60	2341	98 (4.19)	1.70 (1.30–2.23)	< 0.001	1.40 (1.05–1.85) ^a^	0.021
*BMI*						
≤ 25.6	5001	121 (2.42)	1.00	–	1.00	–
> 25.6	1509	91 (6.03)	2.37 (1.81–3.11)	< 0.001	2.01 (1.49–2.70) ^b^	< 0.001
*WC*						
≤ 90.6	4910	117 (2.38)	1.00	–	1.00	–
> 90.6	1600	95 (5.94)	2.44 (1.86–3.19)	< 0.001	1.87 (1.40–2.50) ^c^	< 0.001
*LDL-C*						
≤ 154.3	5695	168 (2.95)	1.00	–	1.00	–
> 154.3	815	44 (5.40)	1.91 (1.37–2.67)	< 0.001	1.56 (1.11–2.19) ^d^	0.010
*CRP*						
≤ 0.86	3030	67 (2.21)	1.00	–	1.00	–
> 0.86	3480	145 (4.17)	2.01 (1.50–2.68)	< 0.001	1.50 (1.11–2.03) ^e^	0.009
*TyG*						
≤ 8.7	4146	102 (2.46)	1.00	–	1.00	–
> 8.7	2364	110 (4.65)	1.90 (1.45–2.49)	< 0.001	1.43 (1.08–1.89) ^f^	0.014

HR, hazard ratio; CI, confidence interval. All models were estimated using Cox proportional hazards regression. Model 1: Unadjusted for any covariates. Model 2: Adjusted for sex, smoking, drinking, SBP, HbA1c, and the listed metabolic variables (age, WC, LDL-C, hs-CRP, TyG) excluding the exposure variable in each panel. a Adjusted for sex, smoking, drinking, WC, SBP, HbA1c, LDL-C, hs-CRP, and TyG; b Adjusted for sex, age, smoking, drinking, SBP, HbA1c, LDL-C, hs-CRP, and TyG; c Adjusted for sex, age, smoking, drinking, SBP, HbA1c, LDL-C, hs-CRP, and TyG; d Adjusted for sex, age, smoking, drinking, WC, SBP, HbA1c, hs-CRP, and TyG; e Adjusted for sex, age, smoking, drinking, WC, SBP, HbA1c, LDL-C, and TyG; f Adjusted for sex, age, smoking, drinking, WC, SBP, HbA1c, LDL-C, and hs-CRP. CMM cardiometabolic multimorbidity, BMI body mass index, WC waist circumference, HDL-C high-density lipoprotein cholesterol, hs-CRP high-sensitivity C-reactive protein, LDL-C low-density lipoprotein cholesterol, SBP systolic blood pressure, TyG triglyceride-glucose.

### Hazard ratios for the risk of incident CMM

Optimal cut‑off values for baseline metabolic parameters were identified using maximally selected rank statistics (Figure S6). The optimal cut‑offs were 60 years for age, 25.6 kg/m² for BMI, 90.6 cm for WC, 154.3 mg/dL for LDL‑C, 0.86 mg/L for hs‑CRP, and 8.7 for the TyG index. Participants with values above these cut‑offs had significantly higher cumulative CMM incidence (all log-rank *P* < 0.001, Figure S7). After multivariable adjustment, older age (> 60 years) was independently associated with increased CMM risk (HR = 1.40, 95% CI: 1.05–1.85, *P* = 0.021). Elevated BMI (> 25.6 kg/m²) showed the strongest association (HR = 2.01, 95% CI: 1.49–2.70, *P* < 0.001), followed by increased WC (> 90.6 cm) (HR = 1.87, 95% CI: 1.40–2.50, *P* < 0.001). Higher LDL-C (> 154.3 mg/dL) (HR = 1.56, 95% CI: 1.11–2.19, *P* = 0.010), elevated hs-CRP (> 0.86 mg/L) (HR = 1.50, 95% CI: 1.11–2.03, *P* = 0.009), and higher TyG index (> 8.7) (HR = 1.43, 95% CI: 1.08–1.89, *P* = 0.014) were each independently associated with a higher risk of incident CMM (Table 2). All models were adjusted for sex, SBP, HbA1c, smoking, drinking, and the metabolic parameters (age, WC, LDL-C, TyG and hs-CRP) not tested as the exposure. Fine‑Gray competing risks models with all‑cause death as the competing event yielded consistent results with the primary Cox analysis (Figure S8 and Table S3). The discriminatory ability of WC for predicting CMM was assessed by ROC analysis (Figure S9; Table S4). The optimal WC cutoff (90.6 cm) showed higher specificity (0.761 vs. 0.362), Youden index (0.209 vs. 0.155), and accuracy (0.751 vs. 0.376).

**Fig. 2:**
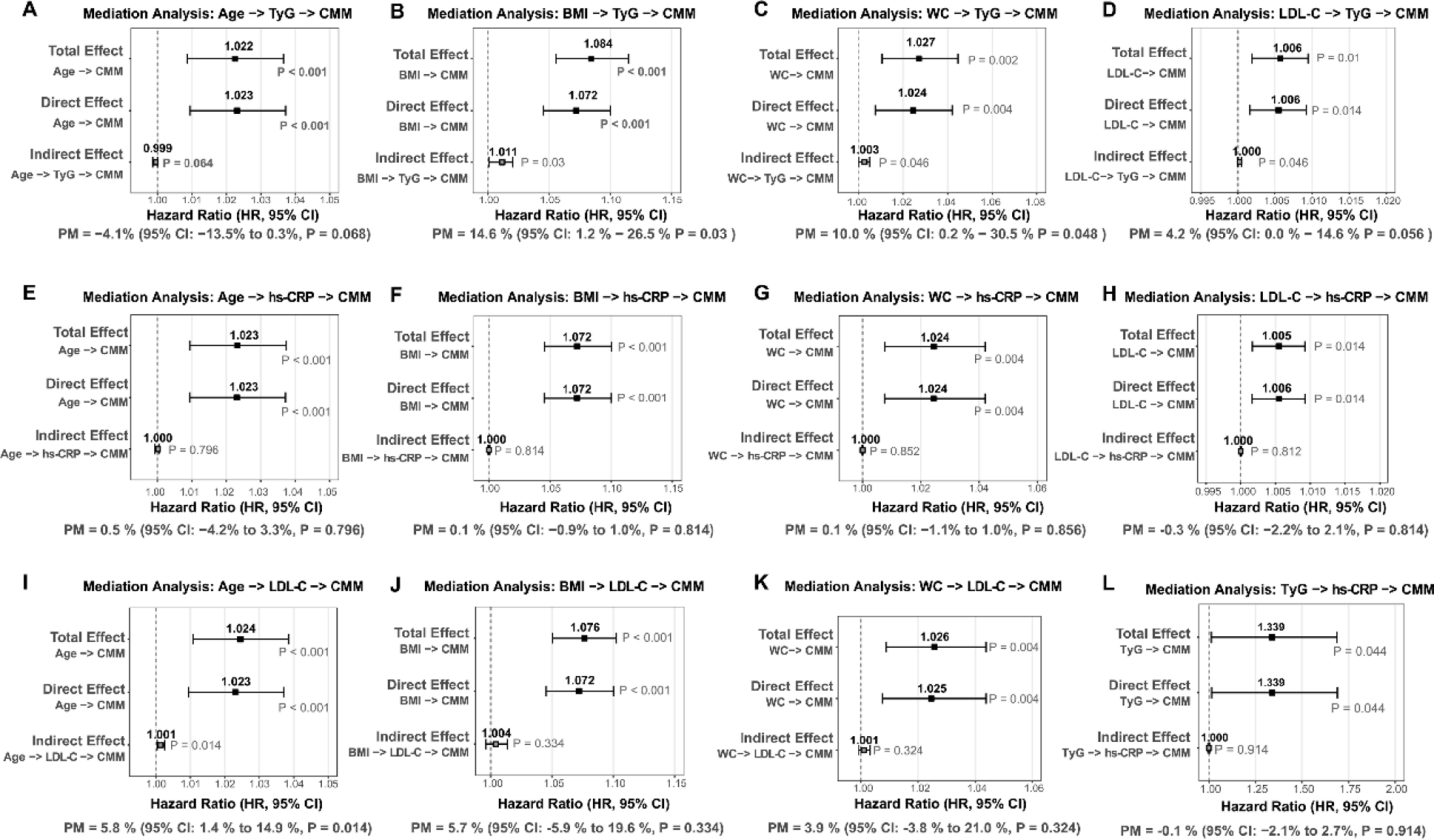
Forest plot of the mediation analysis showing the direct and indirect effects of baseline metabolic parameters on incident CMM. The analysis was performed using a Cox regression-based counterfactual framework implemented in the CMAverse R package. All models estimated the natural direct effect (NDE), natural indirect effect (NIE), and total effect (TE) with adjustment for sex, SBP, and the metabolic covariates (age, WC, LDL-C, hs-CRP, and TyG) not tested as the exposure or mediator. Point estimates and 95% confidence intervals were derived from 1,000 bootstrap resamples using the percentile method. **A–D** Mediating role of the TyG index in the associations of age, BMI, WC, and LDL-C with incident CMM. **E–H & L** Mediating role of hs-CRP in the associations of age, BMI, WC, LDL-C, and TyG index with incident CMM. **I–K** Mediating role of LDL-C in the association of age, BMI, and WC with incident CMM. CMM, cardiometabolic multimorbidity; BMI, body mass index; WC, waist circumference; LDL-C, low-density lipoprotein cholesterol; CRP, C-reactive protein; hs-CRP, high-sensitivity C-reactive protein; TyG, triglyceride-glucose index; NDE, natural direct effect; NIE, natural indirect effect; TE, total effect.

### Interaction analysis

We further examined the combined effects and additive interactions of metabolic parameters on the risk of incident CMM (Table 3). A significant multiplicative interaction was observed for age and hs‑CRP (P for interaction = 0.026), although additive interaction measures (RERI, AP, SI) were not statistically significant. BMI and LDL-C showed a significant multiplicative interaction (P for interaction = 0.039), though additive measures were not significant (RERI − 1.06, 95% CI -2.65 to 0.59; AP -0.44, 95% CI -1.76 to 0.17; SI 0.57, 95% CI 0.13 to 1.33). No significant multiplicative interactions were observed for the remaining pairwise combinations (all P for interaction > 0.05). Despite non‑significant interactions, participants with two concurrent risk factors consistently had the highest CMM incidence and adjusted HRs compared with those with low levels of both factors. For instance, participants with both high age and high BMI had an HR of 2.95 (95% CI: 1.90–4.58) compared with those with low age and low BMI, with an incidence rate of 14.10 per 1,000 person-years

**Table 3:** Combined effects and interaction measures of metabolic parameters on the risk of incident CMM

Variables	Incidence rate (1,000 PY)	HR (95%CI)	*P* for interaction	Interaction measures
*Age & BMI * ^*a*^				
Low age & low BMI	2.95	Reference	0.587	RERI: 0.74 (−0.42, 1.96) AP: 0.25 (−0.16, 0.52) SI: 1.61 (0.76, 4.49)
Low age & high BMI	7.47	1.80 (1.23–2.65)		
High age & low BMI	5.08	1.40 (0.97–2.03)		
High age & high BMI	14.10	2.95 (1.90–4.58)		
*Age & WC* ^*b*^				
Low age & low WC	2.97	Reference	0.748	RERI: 0.18 (−0.93, 1.21) AP: 0.07 (−0.44, 0.39) SI: 1.13 (0.51, 2.80)
Low age & high WC	7.81	1.95 (1.33–2.86)		
High age & low WC	5.07	1.45 (0.99–2.11)		
High age & high WC	11.74	2.58 (1.70–3.93)		
*Age & LDL-C * ^*c*^				
Low age & low LDL-C	3.69	Reference	0.258	RERI: −0.46 (−1.75, 0.64) AP: −0.24 (−1.29, 0.26) SI: 0.68 (0.09, 1.87)
Low age & high LDL-C	7.58	1.90 (1.21−3.00)		
High age & low LDL-C	6.22	1.52 (1.11–2.08)		
High age & high LDL-C	9.56	1.96 (1.19–3.22)		
*Age & hs-CRP * ^*d*^				
Low age & low hs-CRP	2.36	Reference	0.026	RERI: −0.93 (−2.40, 0.04) AP: −0.42 (−1.09, 0.02) SI: 0.57 (0.27, 1.05)
Low age & high hs-CRP	5.98	2.04 (1.35–3.09)		
High age & low hs-CRP	5.84	2.14 (1.31–3.47)		
High age & high hs-CRP	7.27	2.25 (1.45–3.48)		
*Age & TyG * ^*e*^				
Low age & low TyG	3.22	Reference	0.561	RERI: 0.39 (−0.48, 1.17) AP: 0.20 (−0.26, 0.51) SI: 1.64 (−9.60, 12.95)
Low age & high TyG	5.71	1.32 (0.91–1.93)		
High age & low TyG	4.86	1.29 (0.86–1.91)		
High age & high TyG	10.06	2.01 (1.34–2.99)		
*BMI & WC*				
Low BMI & low WC	3.52	Reference	0.485	RERI: 0.64 (−0.59, 1.67) AP: 0.28 (−0.27, 0.69) SI: 2.00 (−20.67, 23.36)
Low BMI & high WC	5.91	1.25 (0.75–2.08)		
High BMI & low WC	5.52	1.40 (0.79–2.47)		
High BMI & high WC	10.52	2.29 (1.66–3.15)		
*BMI & LDL-C*				
Low BMI & low LDL-C	3.24	Reference	0.039	RERI: −1.06 (−2.65, 0.59) AP: −0.44 (−1.76, 0.17) SI: 0.57 (0.13, 1.33)
Low BMI & high LDL-C	7.70	2.10 (1.37–3.23)		
High BMI & low LDL-C	9.11	2.36 (1.70–3.29)		
High BMI & high LDL-C	9.88	2.41 (1.39–4.15)		
*BMI & hs-CRP*				
Low BMI & low hs-CRP	2.94	Reference	0.585	RERI: 0.70 (-0.37, 1.83) AP: 0.26 (-0.15, 0.58) SI: 1.67 (0.61, 7.89)
Low BMI & high hs-CRP	4.59	1.39 (0.96–2.01)		
High BMI & low hs-CRP	5.65	1.65 (0.95–2.87)		
High BMI & high hs-CRP	10.94	2.74 (1.87–4.03)		
*BMI & TyG*				
Low BMI & low TyG	3.07	Reference	0.740	RERI: 0.22 (−0.97, 1.30) AP: 0.08 (−0.37, 0.41) SI: 1.14 (0.59, 2.81)
Low BMI & high TyG	5.26	1.46 (1.01–2.10)		
High BMI & low TyG	7.47	2.10 (1.37–3.22)		
High BMI & high TyG	10.67	2.77 (1.92–4.02)		
*WC & LDL-C*				
Low WC & low LDL-C	3.34	Reference	0.577	RERI: 0.10 (−1.43, 1.70) AP: 0.04 (−0.70, 0.44) SI: 1.06 (0.37, 2.70)
Low WC & high LDL-C	6.31	1.71 (1.08–2.70)		
High WC & low LDL-C	8.46	1.97 (1.42–2.72)		
High WC & high LDL-C	13.03	2.77 (1.70–4.53)		
*WC & hs-CRP*				
Low WC & low hs-CRP	2.89	Reference	0.794	RERI: 0.50 (−0.68, 1.59) AP: 0.19 (−0.27, 0.54) SI: 1.45 (0.56, 7.11)
Low WC & high hs-CRP	4.53	1.44 (0.99–2.10)		
High WC & low hs-CRP	5.86	1.65 (0.96–2.83)		
High WC & high hs-CRP	10.67	2.59 (1.76–3.80)		
*WC & TyG*				
Low WC & low TyG	2.94	Reference	0.373	RERI: −0.10 (−1.44, 0.91) AP: −0.04 (−0.59, 0.31) SI: 0.94 (0.45, 1.99)
Low WC & high TyG	5.35	1.57 (1.08–2.27)		
High WC & low TyG	7.77	2.13 (1.42–3.21)		
High WC & high TyG	10.35	2.60 (1.79–3.76)		
*LDL-C & hs-CRP*				
low LDL-C & low hs-CRP	3.04	Reference	0.325	RERI: −0.38 (−1.81, 1.00) AP: −0.17 (−0.92, 0.36) SI: 0.76 (0.25, 2.62)
low LDL-C & high hs-CRP	5.96	1.60 (1.15–2.24)		
High LDL-C & low hs-CRP	6.36	1.97 (1.07–3.61)		
High LDL-C & high hs-CRP	9.65	2.19 (1.38–3.46)		
*LDL-C & TyG*				
low LDL-C & low TyG	3.44	Reference	0.674	RERI: −0.01 (−1.24, 1.12) AP: −0.01 (−0.75, 0.44) SI: 0.99 (0.26, 4.44)
low LDL-C & high TyG	6.64	1.47 (1.07–2.01)		
High LDL-C & low TyG	6.94	1.68 (1.02–2.78)		
High LDL-C & high TyG	9.92	2.13 (1.35–3.38)		
*hs-CRP & TyG*				
low hs-CRP & low TyG	2.68	Reference	0.658	RERI: 0.01 (−0.99, 0.87) AP: 0.003 (−0.50, 0.37) SI: 1.01 (0.39, 3.57)
low hs-CRP & high TyG	4.96	1.52 (0.93–2.46)		
High hs-CRP & low TyG	4.97	1.59 (1.05–2.39)		
High hs-CRP & high TyG	8.63	2.11 (1.40–3.19)		

Incidence rates are expressed per 1,000 person-years (PY). Hazard ratios (HRs) and 95% confidence intervals (CIs) were estimated using Cox proportional hazards regression models. Additive interaction was assessed using three measures: relative excess risk due to interaction (RERI), attributable proportion due to interaction (AP), and synergy index (SI). RERI > 0, AP > 0, or SI > 1 indicates a positive additive interaction beyond additivity. P values for interaction were calculated by including the product term of the two exposures in the Cox model. All models were adjusted for sex, age, smoking, drinking, SBP, HbA1c, LDL-C, CRP, WC, and TyG index (excluding the variable used for stratification and exposure variable). AP, attributable proportion due to interaction; BMI, body mass index; CI, confidence interval; CRP, C-reactive protein; HbA1c, glycated hemoglobin; HR, hazard ratio; LDL-C, low-density lipoprotein cholesterol; PY, person-years; RERI, relative excess risk due to interaction; SBP, systolic blood pressure; SI, synergy index; TyG, triglyceride-glucose index; WC, waist circumference.

### Mediation effects of metabolic parameters on the risk of incident CMM

Mediation analyses were performed using a Cox-based counterfactual framework with 1,000 bootstrap resamples to explore pathways linking metabolic parameters to incident CMM (Fig. 2 and Figure S10). The TyG index significantly mediated the associations of BMI, WC, and LDL-C with incident CMM. The NIEs for BMI, WC, and LDL-C were all statistically significant, with mediation proportions of 14.6% (95%CI: 1.2%-26.5%), 10.0% (95%CI: 0.2%-30.5%), and 4.2% (95%CI: 0.0%-14.6%), respectively. LDL-C significantly mediated the association between age and incident CMM, with a mediation proportion of 5.8% (95%CI: 1.4%-14.9%)

**Table 4:** Association of K-means risk clusters with incident CMM using Cox proportional hazards regression models

Clusters	Total *N*	Cases *n* (%)	Model 1		Model 2	
			HR (95%CI)	*P* values	HR (95%CI)	*P* values
*Cluster 4*						
Cluster 1	1970	24 (1.22)	1.00	–	1.00	–
Cluster 2	1637	46 (2.81)	2.28 (1.39–3.73)	0.001	2.10 (1.21–3.67)	0.009
Cluster 3	1572	97 (6.17)	5.07 (3.24–7.93)	< 0.001	2.73 (1.50–4.96)	0.001
Cluster 4	1331	45 (3.38)	3.18 (1.94–5.22)	< 0.001	1.34 (0.74–2.44)	0.338
*Cluster 5*						
Cluster 1	1322	20 (1.51)	1.00	–	1.00	–
Cluster 2	1331	42 (3.16)	2.04 (1.20–3.47)	0.009	2.54 (1.39–4.64)	0.002
Cluster 3	1256	78 (6.21)	3.96 (2.42–6.48)	< 0.001	3.06 (1.58–5.93)	0.001
Cluster 4	1042	47 (4.51)	3.49 (2.07–5.89)	< 0.001	1.39 (0.74–2.60)	0.305
Cluster 5	1559	25 (1.60)	1.06 (0.59–1.90)	0.855	1.52 (0.81–2.86)	0.197
*Cluster 6*						
Cluster 1	1238	21 (1.70)	1.00	–	1.00	–
Cluster 2	1057	43 (4.07)	2.80 (1.66–4.72)	< 0.001	1.16 (0.63–2.16)	0.636
Cluster 3	1633	25 (1.53)	0.90 (0.51–1.61)	0.732	1.27 (0.68–2.35)	0.455
Cluster 4	1343	44 (3.28)	1.91 (1.13–3.21)	0.015	2.05 (1.12–3.77)	0.021
Cluster 5	85	1 (1.18)	0.77 (0.10–5.71)	0.796	0.19 (0.01–3.86)	0.279
Cluster 6	1154	78 (6.76)	3.89 (2.40–6.30)	< 0.001	2.77 (1.44–5.36)	0.002
*Cluster 7*						
Cluster 1	920	16 (1.74)	1.00	–	1.00	–
Cluster 2	83	1 (1.20)	0.81 (0.11–6.08)	0.834	0.18 (0.01–3.87)	0.271
Cluster 3	1213	41 (3.38)	2.05 (1.15–3.66)	0.015	2.04 (1.06–3.92)	0.033
Cluster 4	1027	64 (6.23)	3.67 (2.12–6.35)	< 0.001	2.78 (1.44–5.38)	0.002
Cluster 5	1445	24 (1.66)	1.03 (0.55–1.94)	0.929	1.34 (0.70–2.54)	0.378
Cluster 6	942	16 (1.70)	1.13 (0.57–2.27)	0.724	0.80 (0.37–1.70)	0.559
Cluster 7	880	50 (5.68)	4.06 (2.31–7.14)	< 0.001	1.54 (0.79–3.03)	0.207

Data are presented as Hazard Ratios (HRs) with 95% Confidence Intervals (CIs) derived from Cox proportional hazards regression models. Cluster memberships were derived from K-means clustering based on eight standardized cardiometabolic variables: age, BMI, WC, SBP, FPG, LDL-C, hs-CRP, and TyG. Cluster 1 served as the reference group in all clustering solutions. Model 1: Unadjusted. Model 2: Adjusted for age, sex, smoking, drinking SBP, HbA1c, LDL-C, WC, CRP, and TyG. BMI, body mass index; CMM, cardiometabolic multimorbidity; hs-CRP, hs-C-reactive protein; HbA1c, glycated hemoglobin; HR, hazard ratio; LDL-C, low-density lipoprotein cholesterol; SBP, systolic blood pressure; TyG, triglyceride-glucose index; WC, waist circumference.

### Identification of metabolic risk subgroups by K-means clustering and their association with incident CMM risk

To identify underlying metabolic risk patterns, we performed K-means clustering analyses using eight standardized cardiometabolic variables (age, BMI, WC, hs-CRP, TyG, SBP, HDL-C, and FPG). Cluster number and stability were assessed across K = 2–10 using average silhouette width (Table S5), variance explained (Table S6), and adjusted rand index across 20 repeated runs (Table S7). Silhouette width peaked at K = 3 (0.194) but K = 3 merged insulin‑resistant and obese–insulin‑resistant groups, offering limited clinical granularity. Variance explained rose from 18.0% (K = 2) to 52.0% (K = 10), with the largest WCSS reduction at K = 4 (9.56%) and diminishing returns beyond K = 6 (6.20%). Mean ARI was 0.96–0.98 for K = 4–6 versus 0.85 for K = 7. Thus, K = 4 was chosen as the parsimonious solution and K = 6 retained for additional heterogeneity.

**Fig. 3:**
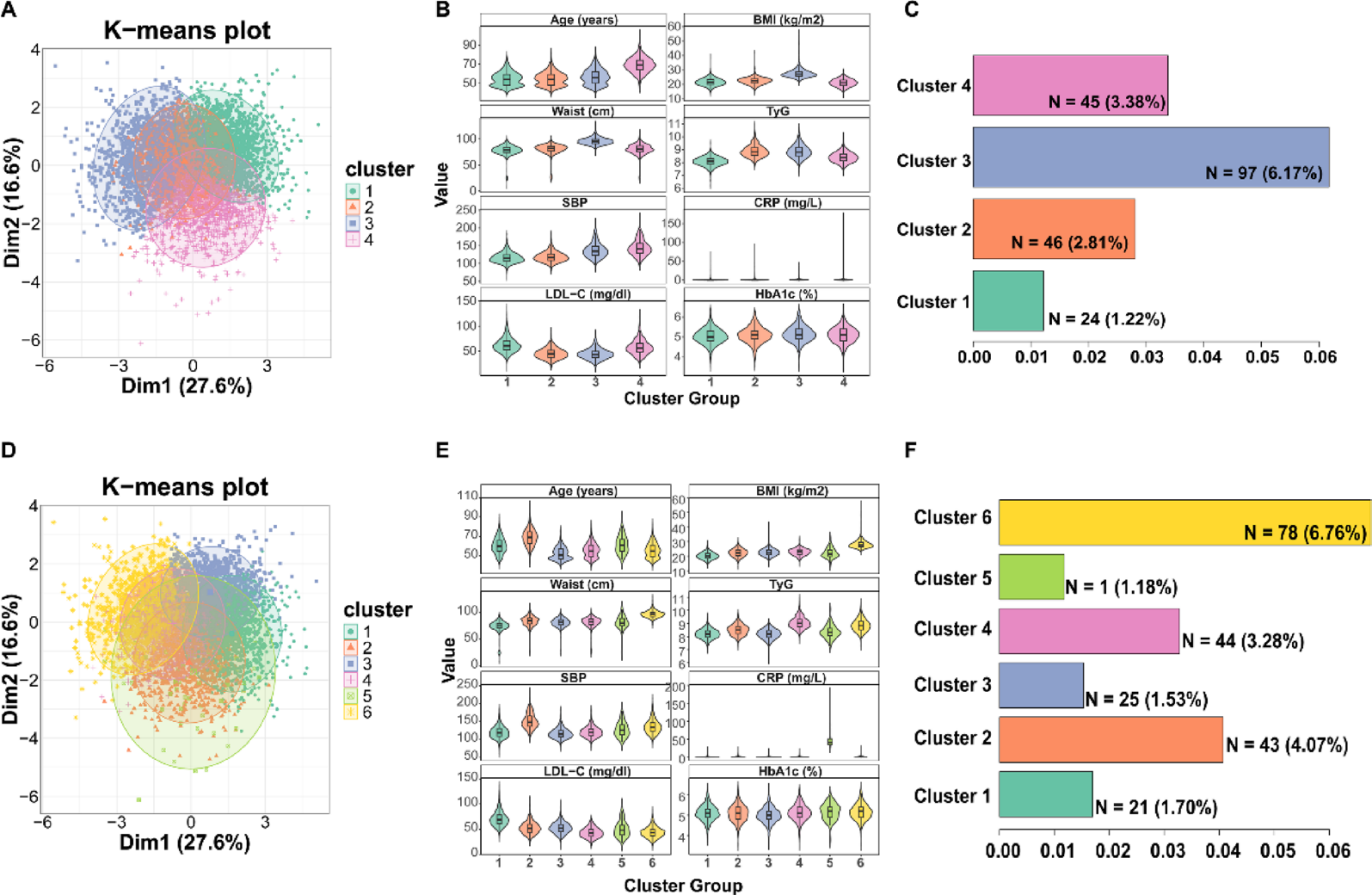
Identification of subgroups by K-means clustering and association with incident CMM. Two-dimensional visualization of participants classified into four **A** and six **D** distinct clusters based on eight standardized cardiometabolic variables: age, BMI, WC, hs-CRP, TyG, SBP, HDL-C, and FPG. **B** Violin plots comparing the levels of individual metabolic indicators across the four clusters. **E** Violin plots comparing the levels of individual metabolic indicators across the six clusters. **C** Bar plot showing the number and proportion of incident CMM events within four cluster. **F** Bar plot showing the number and proportion of incident CMM events within six cluster. P value was calculated using the chi-square test. CMM cardiometabolic multimorbidity, BMI body mass index, WC waist circumference, HDL-C high-density lipoprotein cholesterol, hs-CRP high-sensitivity C-reactive protein, LDL-C low-density lipoprotein cholesterol, SBP systolic blood pressure, TyG triglyceride-glucose; FPG fasting plasma glucose.

**Fig. 4:**
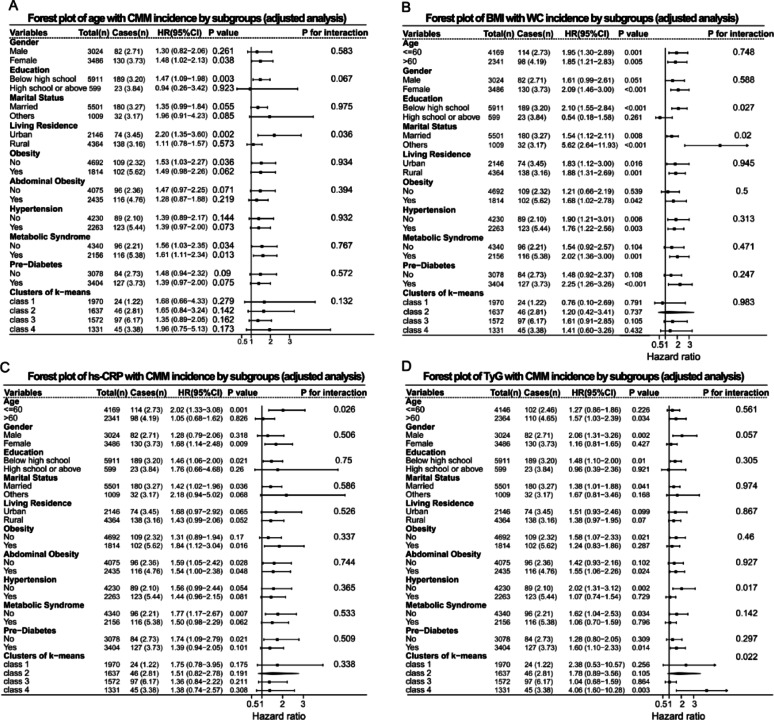
Forest plot of the association of age, WC, hs-CRP and TyG with incident CMM stratified by subgroups. Hazard ratios (HRs) and 95% confidence intervals (CIs) were estimated using Cox proportional hazards regression models. All models were adjusted for sex, age, smoking, drinking, SBP, HbA1c, LDL-C, CRP, WC, and TyG index (excluding the variable used for stratification and exposure variable). Squares represent point estimates of HRs, and horizontal lines represent 95% CIs. The dashed vertical line indicates HR = 1 (null effect). P values for interaction were calculated to assess potential effect modification. **A** Forest plot of age group (≤ 60 vs. >60 years) with CMM incidence by subgroups; **B** Forest plot of WC (≤ 90.6 vs. >90.6 cm) with CMM incidence by subgroups; **C** Forest plot of hs-CRP (≤ 0.86 vs. >0.86 mg/L) with CMM incidence by subgroups; **D** Forest plot of TyG (≤ 8.70 vs. >8.70) with CMM incidence by subgroups. CMM, cardiometabolic multimorbidity; BMI, body mass index; WC, waist circumference; CI, confidence interval; CRP, C-reactive protein; HbA1c, glycated hemoglobin; HR, hazard ratio; LDL-C, low-density lipoprotein cholesterol; SBP, systolic blood pressure; TyG, triglyceride-glucose index.

We evaluated clustering solutions ranging from four to seven clusters (Fig. 3 and Figure S11). The four‑cluster solution revealed distinct metabolic profiles across clusters (Fig. 4 A-C). Cluster 1 (*n* = 1,970, 1.22% CMM events) was characterized by the relatively metabolically healthy profile and served as the reference group. Cluster 2 (*n* = 1,637, 2.81% events) was characterized by elevated TyG and defined as insulin‑resistant. Cluster 3 (*n* = 1,572, 6.17% events) exhibited high BMI, WC, and TyG and was defined as obese–insulin-resistant. Cluster 4 (*n* = 1,331, 3.38% CMM) was characterized by older age and elevated SBP, defined as elderly hypertensive. After adjustment, Clusters 2 (HR 2.10, 95% CI 1.21–3.67) and 3 (HR 2.73, 95% CI 1.50–4.96) remained significantly associated with elevated CMM risk (Table 4).

The five-, six-, and seven-cluster solutions further refined risk stratification (Table 4; Figure S11). In the five ‑cluster solution, the insulin‑resistant (Cluster 2) and obese–insulin‑resistant (Cluster 3) groups showed significantly elevated CMM risk (HR 2.54, 95% CI 1.39–4.64 and HR 3.06, 95% CI 1.58–5.93, respectively). In the six-cluster solution, Clusters 4 and 6, defined respectively as the insulin-resistant (high TyG) and the obese insulin-resistant (high BMI, WC, and TyG), were associated with a significantly elevated CMM risk (Cluster 4: HR = 2.05, 95% CI: 1.12–3.77, *P* = 0.021; Cluster 6: HR = 2.77, 95% CI: 1.44–5.36, *P* = 0.002). In the seven-cluster solution, Clusters 3 and 4, defined respectively as the insulin-resistant (high TyG) and the obese insulin-resistant (high BMI, WC, and TyG), remained significantly associated with CMM risk after adjustment (Cluster 3: HR = 2.04, 95% CI: 1.06–3.92, *P* = 0.033; Cluster 4: HR = 2.78, 95% CI: 1.44–5.38, *P* = 0.002). Across all clustering solutions, clusters with the highest CMM rates consistently showed elevated adiposity (BMI, WC) and insulin resistance (TyG).

To evaluate the stability of the identified metabolic risk phenotypes, radar plots (Figure S12) compared clusters from four- to seven-cluster solutions. Across all solutions, clusters with elevated BMI, WC, and TyG consistently emerged. Fine-Gray competing risks models (Figure S13) further showed that insulin-resistant (high TyG) and the obese insulin-resistant (high BMI, WC, and TyG) clusters consistently had the highest cumulative incidence of CMM (Gray’s test, all *P* < 0.001). These consistent patterns across all clustering solutions validate the robustness of the risk stratification.

### Subgroup analysis for the associations of metabolic parameters with incident CMM risk

To assess the robustness of the primary associations across different population subgroups, we performed stratified analyses for age (> 60 vs. ≤ 60 years), WC (> 90.6 vs. ≤ 90.6 cm), BMI (> 25.6 kg/m² vs. ≤ 25.6 kg/m²), LDL-C (> 154.3 mg/dL vs. ≤ 154.3 mg/dL), hs-CRP (> 0.86 mg/L vs. ≤ 0.86 mg/L) and TyG (> 8.7 vs. ≤ 8.7) by various demographic and clinical factors, including gender, education, marital status, living residence, obesity, abdominal obesity, hypertension, metabolic syndrome, prediabetes, and K-means clusters. The results are presented in forest plots (Fig. 2 and Figure S14). These subgroup analyses demonstrate that the positive associations of metabolic parameters with incident CMM are generally robust across diverse population subgroups. The few observed interactions suggest possible effect modification by social determinants without undermining the overall consistency of the primary findings

### Dose-response relationship between metabolic parameters and the risk of CMM

**Fig. 5:**
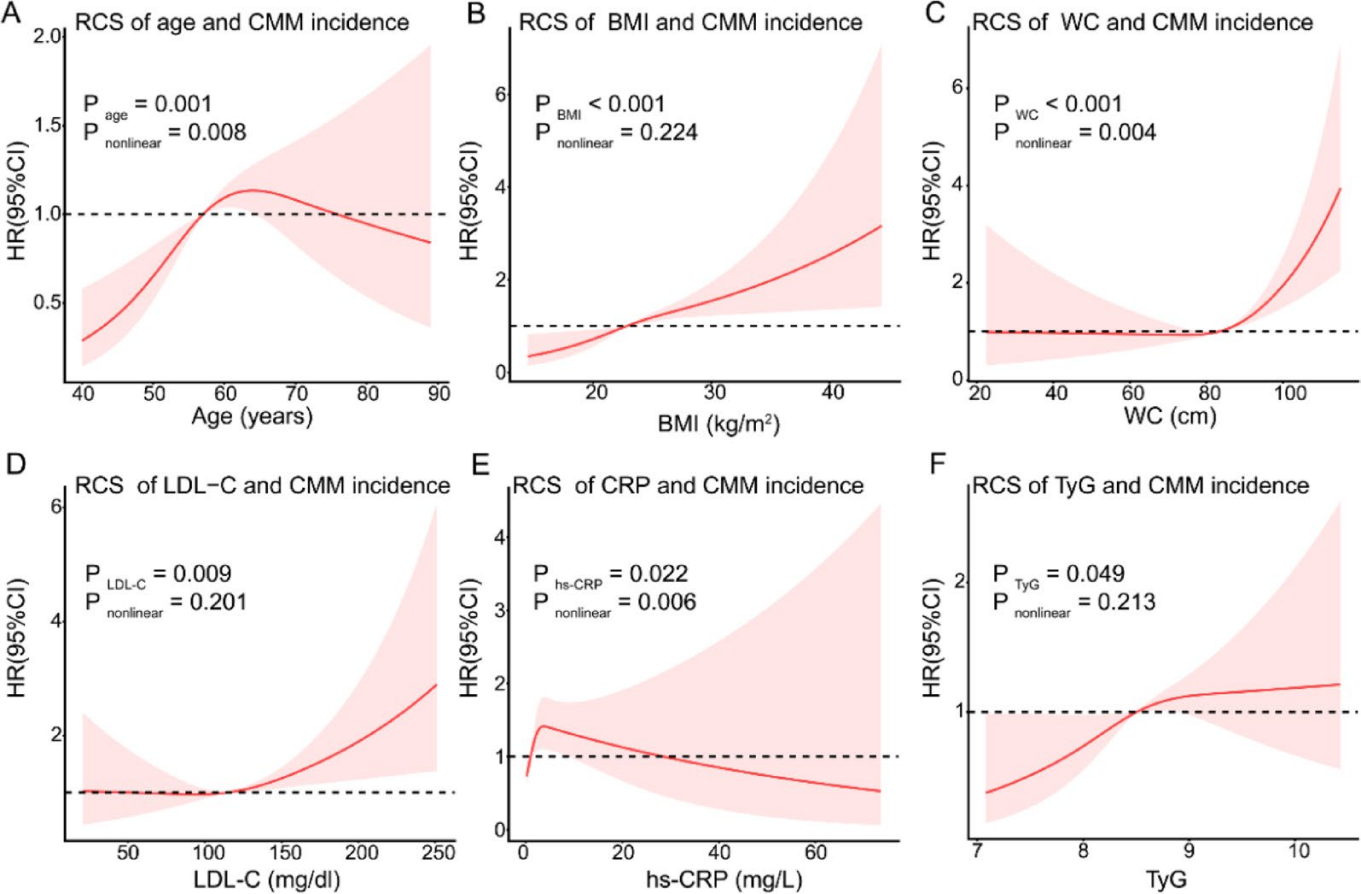
Dose-response relationship between the metabolic parameters and the risk of CMM. Cox proportional hazards regression models with RCS analyses were fitted with three knots at the 10th, 50th, and 90th percentiles, with the reference set at the median, and adjusted for sex, SBP, HbA1c, smoking, drinking, and the metabolic parameters (age, WC, LDL-C, hs-CRP, and TyG) not tested as the exposure. Solid lines represent the estimated hazard ratios (HRs), and shaded areas indicate the 95% confidence intervals. **A** age, **B** BMI, **C** WC, **D** LDL-C, **E** hs-CRP, and **F** TyG. P for overall and P for nonlinearity were derived from likelihood ratio tests. CMM, cardiometabolic multimorbidity; WC, waist circumference; BMI, body mass index; LDL-C, low-density lipoprotein cholesterol; CRP, C-reactive protein; TyG, triglyceride-glucose index; SBP, systolic blood pressure; RCS, restricted cubic spline; HR, hazard ratio.

RCS analyses revealed nonlinear dose-response relationships between metabolic parameters and incident CMM (Fig. 5). CMM risk increased with age up to approximately 55 years and plateaued thereafter (P for age = 0.001, P for nonlinearity = 0.008). BMI (P for BMI < 0.001), LDL-C (P for LDL-C = 0.009), and TyG (P for TyG = 0.049) demonstrated positive linear associations. WC exhibited a J-shaped relationship with risk increasing after ~ 80 cm (P for WC < 0.001, P for nonlinearity = 0.004). hs-CRP showed a nonlinear association with CMM risk rising steeply at lower levels and declining gradually thereafter (P for hs-CRP = 0.022, P for nonlinearity = 0.006). All models were adjusted for sex, SBP, HbA1c, smoking, drinking, and metabolic parameters (age, WC, LDL-C, TyG and hs-CRP) not tested as the exposure. Sensitivity analyses using a multiply imputed dataset (*N* = 11,846) yielded largely consistent findings (Fig. S5), supporting the robustness of our primary conclusions.

### Sensitivity analyses for the associations of metabolic parameters with incident CMM risk

To validate our findings, we conducted sensitivity analyses. First, a 2‑year lag analysis excluded participants who developed CMM within the first 2 years (*n* = 2) (Table S8) or died (*n* = 29) within the first 2 years (Table S10). The Fine-Gray competing risks models were also re-estimated (Table S9 and Table S11). Across all four sensitivity analyses, elevated BMI, WC, LDL‑C, hs-CRP, and TyG remained significantly associated with increased CMM risk, consistent with the primary findings (all *P* < 0.05). Second, we performed multiple imputation and repeated the analyses using the multiply imputed dataset (*N* = 11,846). Baseline characteristics were comparable between the original and imputed datasets (Table S12). After multivariable adjustment, elevated BMI, increased WC, higher LDL-C, elevated hs-CRP, older age, and higher TyG remained significantly associated with incident CMM (all *P* < 0.05, Table S13). Fine-Gray competing risks models produced comparable results (Table S14). Last, PH violations for HbA1c and LDL‑C were addressed by stratified models (Table S15), after which the global PH test was non‑significant (Table S16), and all associations remained consistent with the primary analysis (Table S17).

## Discussion

We evaluated the associations of metabolic parameters with incident CMM. Optimal cut-off values for discriminating CMM risk were identified as follows: age 60 years, BMI 25.6 kg/m², WC 90.6 cm, LDL-C 154.3 mg/dL, hs-CRP 0.86 mg/L, and TyG 8.70. Participants with concurrent exposure to two risk factors consistently exhibited high CMM incidence rates. Mediation analyses further revealed that the TyG index partially mediated the associations of BMI, WC, and LDL-C with CMM. Additionally, K-means clustering based on eight cardiometabolic variables identified distinct metabolic phenotypes, with insulin resistance and obesity emerging as key drivers of CMM progression.

Metabolic parameters provide a critical basis for CMM risk assessment, informing mechanistic exploration and preventive strategies. Obesity, lipid, and glycemic profiles, along with insulin resistance, are established independent predictors of CMM [23–25]. BMI and WC are core anthropometric of CMM risk. Higher BMI is associated with increased risk of diabetes, and heart disease, even within the normal BMI range [26]. Furthermore, studies have shown that WC is a robust predictor of metabolic risk [27, 28]. Identifying optimal cut‑offs for metabolic parameters is essential for screening high‑risk populations. In Chinese populations, the optimal BMI cut‑off for predicting type 2 diabetes was 25.5 kg/m^2^ for men and 24.4 kg/m^2^ for women in those aged 20–39 years [29]. Optimal BMI cut‑offs for cardiometabolic risk in Black South Africans were as low as 22 kg/m^2^ for men and 28 kg/m^2^ for women [30]. Optimal WC cut‑offs for predicting diabetes in rural Chinese populations were reported as 97 cm for men and 98.1 cm for women [31]. Among older Korean adults, optimal WC cut-offs for metabolic syndrome prediction were 89.6 cm in men and 90.5 cm in women aged 65–74 [32]. These findings confirm BMI and WC’s predictive but reveal substantial cut‑off heterogeneity across populations. Our study extends this by establishing cut‑offs for incident CMM in Chinese middle‑aged and older adults. We found that a BMI > 25.6 kg/m^2^ and a WC > 90.6 cm were significantly associated with an increased risk of CMM. The TyG index, a simple surrogate for insulin resistance, has been widely confirmed to predict CVD and CMM risk [33–35], with a cut-off of 8.8 shown to effectively predict diabetes risk [36]. Similarly, we found that a TyG > 8.7 was significantly associated with an increased risk of CMM. hs-CRP as an inflammation-sensitive acute-phase protein, is now recognized as a pivotal marker of chronic low-grade inflammation connecting obesity, insulin resistance, and dyslipidemia to cardiovascular disease. Studies recommend a three-tier stratification with < 1.0 mg/L denoting low risk, 1.0–3.0 mg/L intermediate risk, and > 3.0 mg/L high risk [37]. In a large Iranian cohort, hs‑CRP > 3 mg/dL was associated with elevated TC, LDL‑C, TG, body fat, and blood pressure, independent of obesity and diabetes [38]. LDL-C is recognized as the primary causal factor and therapeutic target for atherosclerotic cardiovascular disease, driving arterial cholesterol deposition, plaque formation, and vascular occlusion [39]. In the present study, hs‑CRP > 0.86 mg/L and LDL‑C > 154.3 mg/dL were significantly associated with increased CMM risk in Chinese middle‑aged and older adults. Together, these results highlight the importance of tailored anthropometric thresholds in CMM prevention, with our identified cut-offs offering clinically practical tools for targeted screening and risk reduction.

CMM commonly arises from the interplay of multiple risk factors, with the co-occurrence of two risk factors often markedly potentiating disease risk. A CHARLS-based study showed that combined assessing insulin resistance and visceral adiposity refines CVD risk stratification [40]. Obesity and hyperuricemia showed a strong additive interaction on NAFLD risk [41]. In Brazilian adolescents, combined elevated TG and WC showed strong additive interaction on diabetes risk [42]. Given the frequent co-occurrence of central adiposity, insulin resistance, and inflammation, we examined their interactions on incident CMM risk. We found that although no pairwise combination reached statistical significance for additive interaction, combined exposure patterns conferred substantially elevated risk. These findings highlight that dual metabolic risk factor exposure markedly amplifies CMM risk even. This supports a shift from single‑parameter screening to integrated metabolic phenotyping in primary prevention to better identify high‑risk individuals.

Mediation analysis elucidates causal pathways and identifies intervention targets beyond traditional association analysis. In a retrospective study, HbA1c mediated up to 27.86% of the coronary heart disease association, indicating that glycemic dysregulation partly underlies the cardiovascular risk of insulin resistance [43]. A UK Biobank study showed that a physical activity related metabolic signature mediated 40.56% of the type 2 diabetes risk and protected against CMM progression (HR 0.58, 95% CI 0.42–0.81) [44]. In our study, the TyG index significantly mediated the associations of BMI, WC, and LDL-C with incident CMM, identifying insulin resistance as a common mediating pathway. Insulin resistance drives cardiovascular damage through endothelial dysfunction, inflammation, and oxidative stress, and is worsened by obesity-induced cytokines in a vicious cycle [45–47]. These findings identify insulin resistance as a central hub linking adiposity and dyslipidemia to CMM, supporting integrated interventions.

Cluster analysis identifies metabolic subtypes beyond single-marker classification, enabling data-driven precision risk stratification, and has been widely applied in metabolic research. For cardiovascular-kidney-metabolic (CKM) syndrome, Yang et al. applied K-means clustering, identifying five subtypes with distinct proteomic and genetic profiles [48]. A three-cohort Asian study validated a clustering model that identified a high cardiometabolic risk cluster and a liver-specific cluster, supporting phenotype-based risk stratification [49]. Study identified five MetS endophenotypes with distinct clinical and genetic profiles in 103,996 UK Biobank participants via unsupervised clustering [50]. Previous studies support the value of clustering in resolving metabolic heterogeneity. Our cluster analysis identified insulin resistant and obese insulin resistant subtypes with elevated CMM risk. These findings support phenotype-based risk stratification and tailored interventions targeting the dominant metabolic abnormality in each subtype.

## Strengths and limitations

Some strengths of this study should be noted. First, this large prospective cohort study provides robust evidence linking adiposity, insulin resistance, and inflammation to CMM risk. Second, this is the first study to evaluate these factors in relation to CMM risk, examining curvilinear relationships, combined effects, and optimal cut-offs. Third, it provides a clinically actionable WC cutoff of 90.6 cm, which outperformed traditional Asian standards. Fourth, K-means clustering identified obesity-dominant and insulin resistance-dominant phenotypes with high CMM risk.

Nevertheless, several limitations warrant consideration. First, CMM was defined using only three conditions (diabetes, heart disease, and stroke), without including peripheral artery disease, chronic kidney disease, hypertension, or dyslipidemia. Heart disease subtypes and diabetes subtypes were not differentiated, which may mask risk heterogeneity. Future studies with larger CMM cohorts and longer follow-up are warranted to apply multistate models or stratified analyses to dissect the differential associations of parameters with specific CMM subtypes and transition patterns. Second, only the TyG index was used to assess insulin resistance, without gold-standard measures such as the Matsuda index. All metabolic indicators were measured only at baseline, precluding trajectory analysis. Future studies are planned to incorporate OGTT-derived indices, explore the dynamic trajectories of WC, BMI, and the TyG index in relation to CMM risk, and investigate the role of low WC while accounting for subclinical disease, unintentional weight loss, and frailty. Third, the interval-based follow-up design resulted in interval-censored CMM onset times, potentially introducing bias. The limited number of CMM events may restrict statistical power, warranting cautious interpretation of the findings. As an observational study, residual confounding and reverse causation remain possible. Residual confounding from unmeasured lifestyle factors (physical activity, diet, medication use) cannot be excluded, and future studies using device-based measurement and validated dietary assessments are warranted. Exclusion of participants with missing covariates may have introduced selection bias. Fourth, the cluster analysis is exploratory and lacks external validation. Silhouette widths were below 0.25, reflecting the continuous nature of cardiometabolic risk without clear biological boundaries. External validation in independent cohorts is needed to confirm the stability and generalizability of these metabolic phenotypes. Finally, the study population was restricted to Chinese adults aged 40 years and older, and the findings may not generalize to younger populations or other ethnic groups. Molecular mechanisms were not explored, and the causal pathways require further validation.

## Conclusion

The identified optimal cut-offs including BMI > 25.6 kg/m², WC > 90.6 cm, TyG > 8.7, and hs-CRP > 0.86 mg/L provide practical screening tools for primary care to identify individuals at high risk of incident CMM. Dual risk factor elevation markedly increases CMM risk, supporting combined metabolic assessment over single-parameter screening. The mediating role of insulin resistance highlights it as a therapeutic target. Clustering consistently identifies insulin-resistant and obese-insulin-resistant subgroups as the highest-risk phenotypes, enabling targeted, phenotype-based prevention to improve clinical outcomes.

## Supplementary Information

Below is the link to the electronic supplementary material.

Supplementary Material 1

## Data Availability

The data that support the findings of this study are publicly available from the China Health and Retirement Longitudinal Study (CHARLS) at http://charls.pku.edu.cn/.

## Abbreviations

CMM: Cardiometabolic multimorbidity
CHARLS: China health and retirement longitudinal study
RCS: Restricted cubic spline(s)
WC: Waist circumference
BMI: Body mass index
TyG: Triglyceride-glucose
hs-CRP: C-reactive protein
HR: Hazard ratio
CI: Confidence interval
SHR: Subdistribution hazard ratio
RERI: Relative excess risk due to interaction
TG: Triglycerides
TC: Total cholesterol
LDL-C: Low-density lipoprotein cholesterol
HDL-C: High-density lipoprotein cholesterol
FPG: Fasting plasma glucose
HbA1c: Hemoglobin A1c
SBP: Systolic blood pressure
DBP: Diastolic blood pressure
SI: Synergy index
AP: Attribu.able proportion
NDE: Natural direct effect
NIE: Natural indirect effect
TE: Total effect
IPCW: Inverse probability of censoring weighting
PPV: Positive predictive value
NPV: Negative predictive value
MAR: Missing at random
MICE: Multiple imputation by chained equations
VIF: Variance inflation factor
FDR: False discovery rate
ARI: Adjusted rand index
WCSS: Within-cluster sum of squares
MetS: Metabolic syndrome
CIF: Cumulative incidence function

## References

[1] Barnett K, Mercer SW, Norbury M, Watt G, Wyke S, Guthrie B. Epidemiology of multimorbidity and implications for health care, research, and medical education: a cross-sectional study. Lancet. 2012;380(9836):37–43. 22579043 10.1016/S0140-6736(12)60240-2

[2] Han Y, Hu Y, Yu C, Guo Y, Pei P, Yang L, Chen Y, Du H, Sun D, Pang Y, Chen N, Clarke R, Chen J, Chen Z, Li L, Lv J. Lifestyle, cardiometabolic disease, and multimorbidity in a prospective Chinese study. Eur Heart J. 2021;42(34):3374–84. 34333624 10.1093/eurheartj/ehab413 PMC8423468

[3] Dove A, Marseglia A, Shang Y, Grande G, Vetrano DL, Laukka EJ, Fratiglioni L, Xu W. Cardiometabolic multimorbidity accelerates cognitive decline and dementia progression. Alzheimers Dement. 2023;19(3):821–30. 35708183 10.1002/alz.12708

[4] Dove A, Guo J, Marseglia A, Fastbom J, Vetrano DL, Fratiglioni L, Pedersen NL, Xu W. Cardiometabolic multimorbidity and incident dementia: the Swedish twin registry. Eur Heart J. 2023;44(7):573–82. 36577740 10.1093/eurheartj/ehac744 PMC9925275

[5] Otieno P, Asiki G, Aheto JMK, Wilunda C, Sanya RE, Wami W, Mwanga D, Agyemang C. Cardiometabolic multimorbidity associated with moderate and severe disabilities: results from the study on global ageing and adult health (SAGE) Wave 2 in Ghana and South Africa. Glob Heart. 2023;18(1):9. 36874442 10.5334/gh.1188 PMC9983501

[6] Canoy D, Tran J, Zottoli M, Ramakrishnan R, Hassaine A, Rao S, Li Y, Salimi-Khorshidi G, Norton R, Rahimi K. Association between cardiometabolic disease multimorbidity and all-cause mortality in 2 million women and men registered in UK general practices. BMC Med. 2021;19(1):258. 34706724 10.1186/s12916-021-02126-x PMC8555122

[7] Kim MS, Kim WJ, Khera AV, Kim JY, Yon DK, Lee SW, Shin JI, Won HH. Association between adiposity and cardiovascular outcomes: an umbrella review and meta-analysis of observational and Mendelian randomization studies. Eur Heart J. 2021;42(34):3388–403. 34333589 10.1093/eurheartj/ehab454 PMC8423481

[8] Ross R, Neeland IJ, Yamashita S, Shai I, Seidell J, Magni P, Santos RD, Arsenault B, Cuevas A, Hu FB, Griffin BA, Zambon A, Barter P, Fruchart JC, Eckel RH, Matsuzawa Y, Després JP. Waist circumference as a vital sign in clinical practice: a consensus statement from the IAS and ICCR Working Group on Visceral Obesity. Nat Rev Endocrinol. 2020;16(3):177–89. 32020062 10.1038/s41574-019-0310-7 PMC7027970

[9] Busetto L, Dicker D, Frühbeck G, Halford JCG, Sbraccia P, Yumuk V, Goossens GH. A new framework for the diagnosis, staging and management of obesity in adults. Nat Med. 2024;30(9):2395–99. 38969880 10.1038/s41591-024-03095-3

[10] Kojta I, Chacińska M, Błachnio-Zabielska A, Obesity. Bioactive Lipids, and Adipose Tissue Inflammation in Insulin Resistance. Nutrients2020;12(5). 10.3390/nu12051305 PMC7284998 32375231

[11] Wu H, Ballantyne CM. Metabolic inflammation and insulin resistance in obesity. Circ Res. 2020;126(11):1549–64. 32437299 10.1161/CIRCRESAHA.119.315896 PMC7250139

[12] Tian Z, Yang L, Li Y, Huang Y, Yang J, Xue F. Associations of different insulin resistance-related indices with the incidence and progression trajectory of cardiometabolic multimorbidity: a prospective cohort study from UK biobank. Cardiovasc Diabetol. 2025;24(1):257. 40533754 10.1186/s12933-025-02819-0 PMC12175334

[13] Cheng W, Du Z, Lu B. Chronic low-grade inflammation associated with higher risk and earlier onset of cardiometabolic multimorbidity in middle-aged and older adults: a population-based cohort study. Sci Rep. 2024;14(1):22635. 39349699 10.1038/s41598-024-72988-7 PMC11442589

[14] Wan B, Wang S, Hu S, Han W, Qiu S, Zhu L, Ruan L, Wei Y, Xu J. The comprehensive effects of high-sensitivity C-reactive protein and triglyceride glucose index on cardiometabolic multimorbidity. Front Endocrinol (Lausanne). 2025;16:1511319. 40235659 10.3389/fendo.2025.1511319 PMC11996647

[15] Chen X, Crimmins E, Hu PP, Kim JK, Meng Q, Strauss J, Wang Y, Zeng J, Zhang Y, Zhao Y. Venous blood-based biomarkers in the China Health and retirement longitudinal study: rationale, design, and results from the 2015 wave. Am J Epidemiol. 2019;188(11):1871–77. 31364691 10.1093/aje/kwz170 PMC6825825

[16] Ren Q, Huang Y, Liu Q, Chu T, Li G, Wu Z. Association between triglyceride glucose-waist height ratio index and cardiovascular disease in middle-aged and older Chinese individuals: a nationwide cohort study. Cardiovasc Diabetol. 2024;23(1):247. 38992634 10.1186/s12933-024-02336-6 PMC11241990

[17] Zhou BF. Effect of body mass index on all-cause mortality and incidence of cardiovascular diseases–report for meta-analysis of prospective studies open optimal cut-off points of body mass index in Chinese adults. Biomed Environ Sci. 2002;15(3):245–52. 12500665

[18] Xi B, Liang Y, He T, Reilly KH, Hu Y, Wang Q, Yan Y, Mi J. Secular trends in the prevalence of general and abdominal obesity among Chinese adults, 1993–2009. Obes Rev. 2012;13(3):287–96. 22034908 10.1111/j.1467-789X.2011.00944.x PMC3276709

[19] Shimamoto K, Ando K, Fujita T, Hasebe N, Higaki J, Horiuchi M, Imai Y, Imaizumi T, Ishimitsu T, Ito M, Ito S, Itoh H, Iwao H, Kai H, Kario K, Kashihara N, Kawano Y, Kim-Mitsuyama S, Kimura G, Kohara K, Komuro I, Kumagai H, Matsuura H, Miura K, Morishita R, Naruse M, Node K, Ohya Y, Rakugi H, Saito I, Saitoh S, Shimada K, Shimosawa T, Suzuki H, Tamura K, Tanahashi N, Tsuchihashi T, Uchiyama M, Ueda S, Umemura S. The Japanese society of hypertension guidelines for the management of hypertension (JSH 2014). Hypertens Res. 2014;37(4):253–390. 24705419 10.1038/hr.2014.20

[20] Ko GT, Cockram CS, Chow CC, Yeung VT, Chan WB, So WY, Chan NN, Chan JC. Metabolic syndrome by the international diabetes federation definition in Hong Kong Chinese. Diabetes Res Clin Pract. 2006;73(1):58–64. 16406127 10.1016/j.diabres.2005.11.009

[21] 2. Diagnosis and classification of diabetes: standards of care in diabetes-2024. Diabetes Care. 2024;47(Suppl 1):S20–42. 38078589 10.2337/dc24-S002 PMC10725812

[22] Zhao Y, Hu Y, Smith JP, Strauss J, Yang G. Cohort profile: the China Health and Retirement Longitudinal Study (CHARLS). Int J Epidemiol. 2014;43(1):61–8. 23243115 10.1093/ije/dys203 PMC3937970

[23] Du J, Man Z, Bai X, Luo D, Guo Q, Wang Y, Yan Y, Su X, Zheng W. Association of C-reactive protein-triglycerides-glucose index-waist to height ratio and cardiometabolic multimorbidity in middle-aged and older adults: a nationwide cohort study. Cardiovasc Diabetol2026;25(1). 10.1186/s12933-026-03151-x PMC13067722 41923235

[24] Lai H, Tu Y, Liao C, Zhang S, He L, Li J. Joint assessment of abdominal obesity and non-traditional lipid parameters for primary prevention of cardiometabolic multimorbidity: insights from the China health and retirement longitudinal study 2011–2018. Cardiovasc Diabetol. 2025;24(1):109. 40057762 10.1186/s12933-025-02667-y PMC11890515

[25] Zheng W, Man Z, Ren Y, Li Y, Zhu X, Wang L, Zhang X, Hu G, Cao Y. Association of the triglyceride glucose-Chinese visceral adiposity index with incident cardiometabolic multimorbidity in middle-aged and older adults: a nationwide prospective cohort study. Cardiovasc Diabetol2026;25(1). 10.1186/s12933-026-03091-6 PMC12964883 41639722

[26] Lin H, Xiao N, Lin S, Liu M, Liu GG. Associations of hypertension, diabetes and heart disease risk with body mass index in older Chinese adults: a population-based cohort study. BMJ Open. 2024;14(7):e083443. 38986550 10.1136/bmjopen-2023-083443 PMC11268030

[27] Zhang L, Liu Z, Si D, Yang H, Fan X, Yan L, Liu J, Li X, Hu J, Wu J. Comparative predictive values of anthropometric indices for cardiometabolic multimorbidity in middle-aged and older adults: a prospective study from the CHARLS study. BMC Public Health. 2025;26(1):333. 41437013 10.1186/s12889-025-26060-2 PMC12838432

[28] Wei S, Yu S, Qian C, Lan Y, Xufeng H. Unveiling the relationship between stress-hyperglycemia ratio and cardiometabolic multimorbidity risk using interpretable machine learning. Eur J Med Res. 2026;31(1):284. 41555416 10.1186/s40001-026-03906-y PMC12895724

[29] Ma H, Wu X, Guo X, Yang J, Ma X, Lv M, Li Y. Optimal body mass index cut-off points for prediction of incident diabetes in a Chinese population. J Diabetes. 2018;10(12):926–33. 29802755 10.1111/1753-0407.12785

[30] Kruger HS, Schutte AE, Walsh CM, Kruger A, Rennie KL. Body mass index cut-points to identify cardiometabolic risk in black South Africans. Eur J Nutr. 2017;56(1):193–202. 26458965 10.1007/s00394-015-1069-9

[31] Hamzeh B, Bagheri A, Pasdar Y, Darbandi M, Rezaeian S, Najafi F, Moradinazar M. Predicting metabolic syndrome by anthropometric measures among adults 35–65 years in the west of Iran; a cross sectional study from an Iranian RaNCD cohort data. Diabetes Metab Syndr2020 Sep-Oct;14(5):1293–98. 10.1016/j.dsx.2020.07.017 32755824

[32] So ES, Yoo KS. Waist circumference cutoff points for central obesity in the Korean elderly population. J Appl Gerontol. 2015;34(1):102–17. 25548090 10.1177/0733464812464428

[33] Li C, Luo X, Chen Y, Lin J, Gu S. Predictive value of an integrated insulin resistance and lipometabolic score for cardiometabolic multimorbidity in older adults: a UK cohort study. Cardiovasc Diabetol2026;25(1). 10.1186/s12933-025-03064-1 PMC12947332 41639900

[34] Zhang F, Zhang B, Huang X, Wang Z, Wang J, Lan H, Xie G, Wang W, Zou Y, Wang C. Comparison of the TyG index, TyG-traditional obesity indices, and TyG-novel obesity indices: does increased complexity help in predicting cardiometabolic multimorbidity? Lipids Health Dis. 2025;25(1):33. 41470001 10.1186/s12944-025-02836-8 PMC12860121

[35] Liao C, Liu H, Xu S, Ling Z, Zhuo Y, Huang G, Lin W, Zhang Z. Associations of TyG-derived indices with cardiometabolic multimorbidity risk in community-dwelling older adults: a longitudinal analysis based on the gold-health cohort. Nutrients2026;18(6). 10.3390/nu18060985 PMC13029052 41901160

[36] Lee DY, Lee ES, Kim JH, Park SE, Park CY, Oh KW, Park SW, Rhee EJ, Lee WY. Predictive value of triglyceride glucose index for the risk of incident diabetes: a 4-year retrospective longitudinal study. PLoS ONE. 2016;11(9):e0163465. 27682598 10.1371/journal.pone.0163465 PMC5040250

[37] Płóciniczak A, Dzięgielewska-Gęsiak S, Brożek A, Blacha A, Nowicki M, Formanowicz D. High sensitivity C-reactive protein as a cardiovascular risk marker in independent community-living elderly persons. J Biol Regul Homeost Agents2018 Sep-Oct;32(5):1199–204. 30334413

[38] Ebrahimi M, Heidari-Bakavoli AR, Shoeibi S, Mirhafez SR, Moohebati M, Esmaily H, Ghazavi H, Saberi Karimian M, Parizadeh SM, Mohammadi M, Mohaddes Ardabili H, Ferns GA, Ghayour-Mobarhan M. Association of serum hs-CRP levels with the presence of obesity, diabetes mellitus, and other cardiovascular risk factors. J Clin Lab Anal. 2016;30(5):672–6. 26857805 10.1002/jcla.21920 PMC6807047

[39] Humphrey R, Bowman L. LDL-cholesterol lowering therapies: an update. Br J Hosp Med (Lond). 2026;87(4):53219. 42052993 10.31083/BJHM53219

[40] Yang Y, Li S, Ren Q, Qiu Y, Pan M, Liu G, Zheng R, An Z, Li S. The interaction between triglyceride-glucose index and visceral adiposity in cardiovascular disease risk: findings from a nationwide Chinese cohort. Cardiovasc Diabetol. 2024;23(1):427. 39604987 10.1186/s12933-024-02518-2 PMC11603997

[41] Zhang S, Du T, Li M, Lu H, Lin X, Yu X. Combined effect of obesity and uric acid on nonalcoholic fatty liver disease and hypertriglyceridemia. Med (Baltim). 2017;96(12):e6381. 10.1097/MD.0000000000006381 PMC5371466 28328829

[42] Deusdará R, de Moura Souza A, Szklo M. Positive additive and multiplicative interactions among clustered components of metabolic syndrome with type 2 diabetes mellitus among brazilian adolescent students. Nutrients2022;14(21). 10.3390/nu14214640 PMC9655281 36364903

[43] Sun Y, Huang J, Yu X, Meng J, Qiu G. Association of the triglyceride glucose index and its related indices with coronary artery disease risk: mediation by glycated haemoglobin. Front Endocrinol (Lausanne). 2026;17:1809007. 42039131 10.3389/fendo.2026.1809007 PMC13106115

[44] Wang J, Zheng Y, Jiang Y, Suo C, Zhang T, Chen X, Xu K. Association between physical activity-related metabolic signature and cardiometabolic diseases and multimorbidity: a cohort study from UK biobank. Prev Med. 2025;191:108211. 39708996 10.1016/j.ypmed.2024.108211

[45] Cersosimo E, DeFronzo RA. Insulin resistance and endothelial dysfunction: the road map to cardiovascular diseases. Diabetes Metab Res Rev2006 Nov-Dec;22(6):423–36. 10.1002/dmrr.634 16506274

[46] Shoelson SE, Lee J, Goldfine AB. Inflammation and insulin resistance. J Clin Invest. 2006;116(7):1793–801. 16823477 10.1172/JCI29069 PMC1483173

[47] Mastrototaro L, Roden M. Insulin resistance and insulin sensitizing agents. Metabolism. 2021;125:154892. 34563556 10.1016/j.metabol.2021.154892

[48] Yang M, Su C, Chang X, Wang G, Liu J. Data-driven cluster analysis identifies distinct types of cardiovascular-kidney-metabolic syndrome. Eur J Prev Cardiol2025 Nov 6. 10.1093/eurjpc/zwaf708 41206602

[49] Zhou XD, Song SJ, Guo CY, Chen QF, Lai-Hung Wong G, Ye TR, Bee Goh GB, Bee YM, Lian LY, Cheuk-Fung Yip T, Che-To Lai J, Lei SY, Liu WY, Fan RC, Hong CL, Targher G, Byrne CD, Marot G, Raverdy V, Pattou F, Zheng Lim DY, Wai-Sun Wong V, Zheng MH. Validation of a data-driven clustering model for MASLD: evidence from three large-scale Asian cohorts. JHEP Rep. 2026;8(1):101645. 41438343 10.1016/j.jhepr.2025.101645 PMC12721048

[50] Lim AMW, Lim EU, Chen PL, Fann CSJ. Unsupervised clustering identified clinically relevant metabolic syndrome endotypes in UK and Taiwan Biobanks. iScience2024;27(7):109815. 10.1016/j.isci.2024.109815 PMC11260869 39040048

