# Supplementary material for: Effects of adiposity, insulin resistance, and inflammation on cardiometabolic multimorbidity: insights from interaction analyses and metabolic phenotyping in the China health and retirement longitudinal study 2011–2018
Source: Cardiovasc Diabetol. 2026 Jun 3;25:224. doi: 10.1186/s12933-026-03234-9 (PMC13459958; doi:10.1186/s12933-026-03234-9)
Supplement: Supplementary file 1 [file 12933_2026_3234_MOESM1_ESM.pdf]

Supplemental materials

Figure S1. Flowchart of the analytical sample selection from the CHARLS 2011–2018 cohort.

Figure S2. Determination of the optimal number of clusters for K-means clustering using the elbow method.

Figure S3. Distribution of missing data across baseline variables.

Figure S4. Heatmap of correlation matrix among independent variables.

Figure S5. Sensitivity analysis: dose-response relationships between baseline metabolic parameters and incident CMM in the multiply imputed dataset (N = 11,846)

Figure S6. Determination of the optimal cut-off value for continuous variables using maximally selected rank statistics for CMM incidence risk.

Figure S7. Kaplan-Meier curves for incident CMM stratified by optimal cut-off values derived from maximally selected rank statistics.

Figure S8. Cumulative incidence function (CIF) curves for incident CMM estimated using the Fine-Gray competing risks model.

Figure S9. ROC curve of WC for predicting CMM and comparison of predictive performance across different WC thresholds

Figure S10. Path diagrams of the mediation analysis illustrating the direct and indirect effects of metabolic risk factors on CMM.

Figure S11. Identification of subgroups by K-means clustering and their association with incident CMM.

Figure S12. Radar plots of metabolic profiles across K-means clustering solutions (K = 4 to 7)

Figure S13. Cumulative incidence of CMM by K-means clusters across different clustering solutions

Figure S14. Forest plot of the association of BMI and LDL-C with incident CMM stratified by subgroups.

Table S1. Baseline characteristics of included and excluded participants

Table S2. Variance inflation factors (VIF) across models with metabolic indicators

Table S3. Sensitivity analysis of the association between variables and incident CMM using the Fine-Gray competing risks model.

Table S4. Comparison of predictive performance between the study-identified optimal cutoff and the Asian standard for WC in predicting CMM

Table S5. Average silhouette width across K-means clustering solutions (K = 2 to 10)

Table S6. Within-cluster sum of squares and variance explained across K-means clustering solutions (K = 2 to 10)

Table S7. Stability of K-means clustering solutions (K = 4 to 7) assessed by the adjusted Rand index across 20 repeated runs

Table S8. Sensitivity analysis: associations of baseline metabolic parameters with risk of incident CMM after excluding events occurring within the first 2 years of follow-up (2-year lag analysis).

Table S9. Sensitivity analysis: Fine-Gray competing risks model for the associations of baseline metabolic parameters with risk of incident CMM after excluding events occurring within the first 2 years of follow-up (2-year lag analysis).

Table S10. Sensitivity analysis: associations of baseline metabolic parameters with risk of incident CMM and death after excluding events occurring within the first 2 years of follow-up (2-year lag analysis).

Table S11. Sensitivity analysis: Fine-Gray competing risks model for the associations of baseline metabolic parameters with risk of incident CMM and death after excluding events occurring within the first 2 years of follow-up (2-year lag analysis).

Table S12. Comparison of baseline characteristics between the original dataset and the multiply imputed dataset.

Table S13. Sensitivity analysis of the association between variables and incident CMM using the cox regression model using the multiply imputed dataset.

Table S14. Sensitivity analysis of the association between variables and incident CMM using the Fine-Gray competing risks model using the multiply imputed dataset.

Table S15. Assessment of the proportional hazard's assumption using scaled

Schoenfeld residuals across six Cox models

Table S16. Global tests in multivariable-adjusted Cox models stratified by LDL-C and HbA1c across six primary exposures models

Table S17. Associations of metabolic indicators with incident CMM: Cox proportional hazards models stratified by LDL-C and HbA1c following Schoenfeld residuals testing

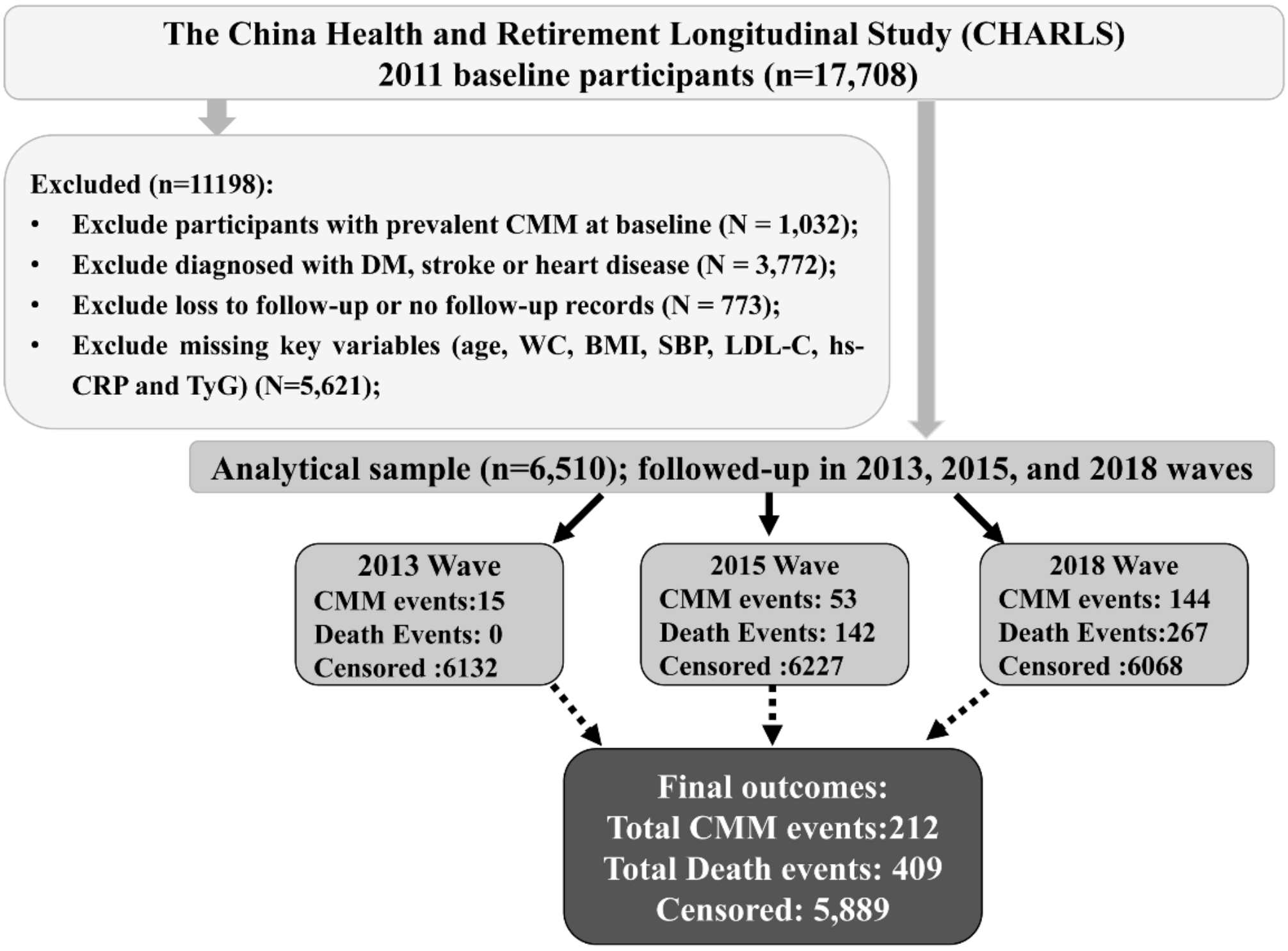

Figure S1. Flowchart of the analytical sample selection from the CHARLS 2011–2018 cohort.

The flowchart illustrates the stepwise exclusion of participants from the 2011 baseline survey through the final analytical sample. Of the 17,708 participants enrolled at baseline, 373 were excluded due to missing baseline CMM data, 680 due to prevalent CMM at baseline, 1,260 due to missing follow-up time, and 5,036 due to other reasons (including missing covariates such as waist circumference, blood biomarkers, and other exclusion criteria). The remaining 10,359 participants constituted the analytical sample and were followed across three waves (2013, 2015, and 2018). Incident CMM was defined as the first occurrence of at least two of the following conditions during follow-up: diabetes mellitus, heart disease, and stroke.

Abbreviations: CHARLS, China Health and Retirement Longitudinal Study; CMM, cardiometabolic multimorbidity.

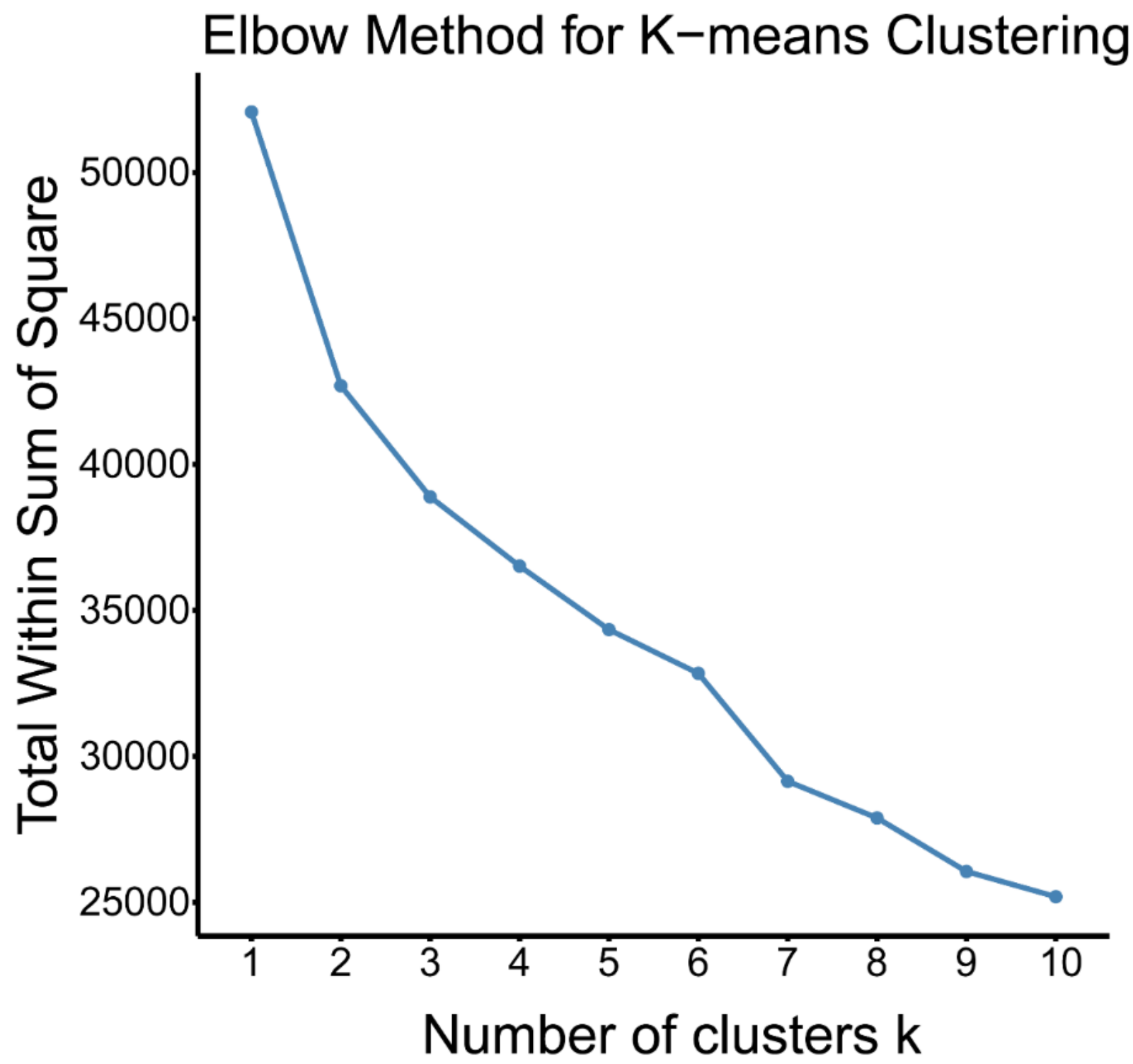

Figure S2. Determination of the optimal number of clusters for K-means clustering using the elbow method.

The elbow plot shows the total within-cluster sum of squares (WCSS) for K-means clustering solutions ranging from K = 1 to 10. The optimal number of clusters is identified at the point where the rate of WCSS reduction markedly decreases (the "elbow"). Based on this plot, K = 4 to 6 were considered as candidate solutions, and were selected for primary presentation after further evaluation of clinical interpretability and cluster stability. K-means clustering was performed with 50 random initializations using eight standardized cardiometabolic variables: age, BMI, WC, hs-CRP, TyG, SBP, HDL-C, and FPG.

Abbreviations: WCSS, within-cluster sum of squares; BMI, body mass index; WC, waist circumference; hs-CRP, high-sensitivity C-reactive protein; TyG, triglyceride-glucose index; SBP, systolic blood pressure; HDL-C, high-density lipoprotein cholesterol; FPG, fasting plasma glucose.

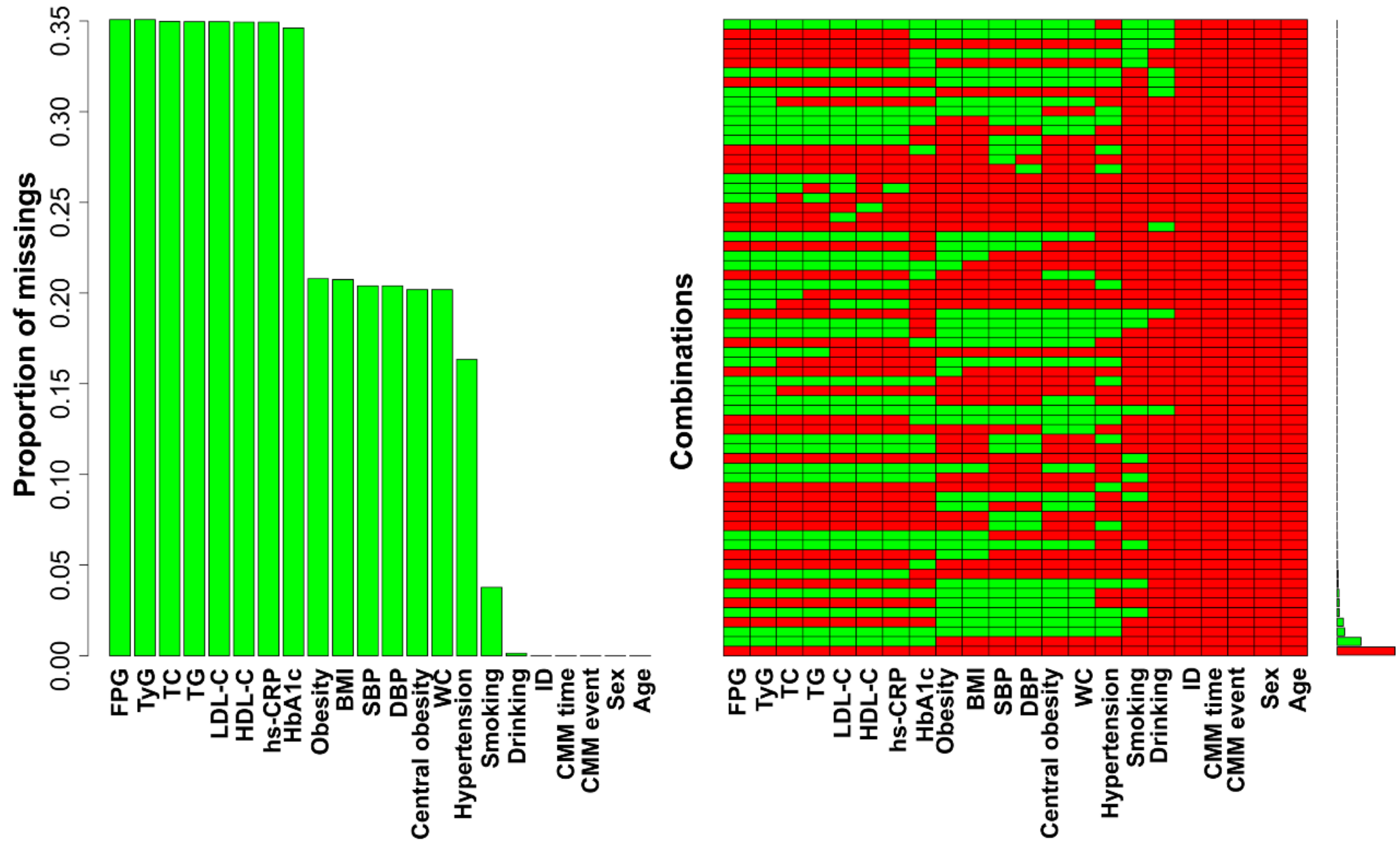

Figure S3. Distribution of missing data across baseline variables.

The bar chart shows the proportion of missing values for each variable in the original dataset (N = 11,846).

Abbreviations: CMM cardiometabolic multimorbidity, BMI body mass index, WC waist circumference, HDL-C high-density lipoprotein cholesterol, hs-CRP high-sensitivity C-reactive protein, LDL-C low-density lipoprotein cholesterol, SBP systolic blood pressure, TyG triglyceride-glucose; FPG fasting plasma glucose; TC total Cholesterol; TG triglycerides; HDL-C high-density lipoprotein cholesterol.

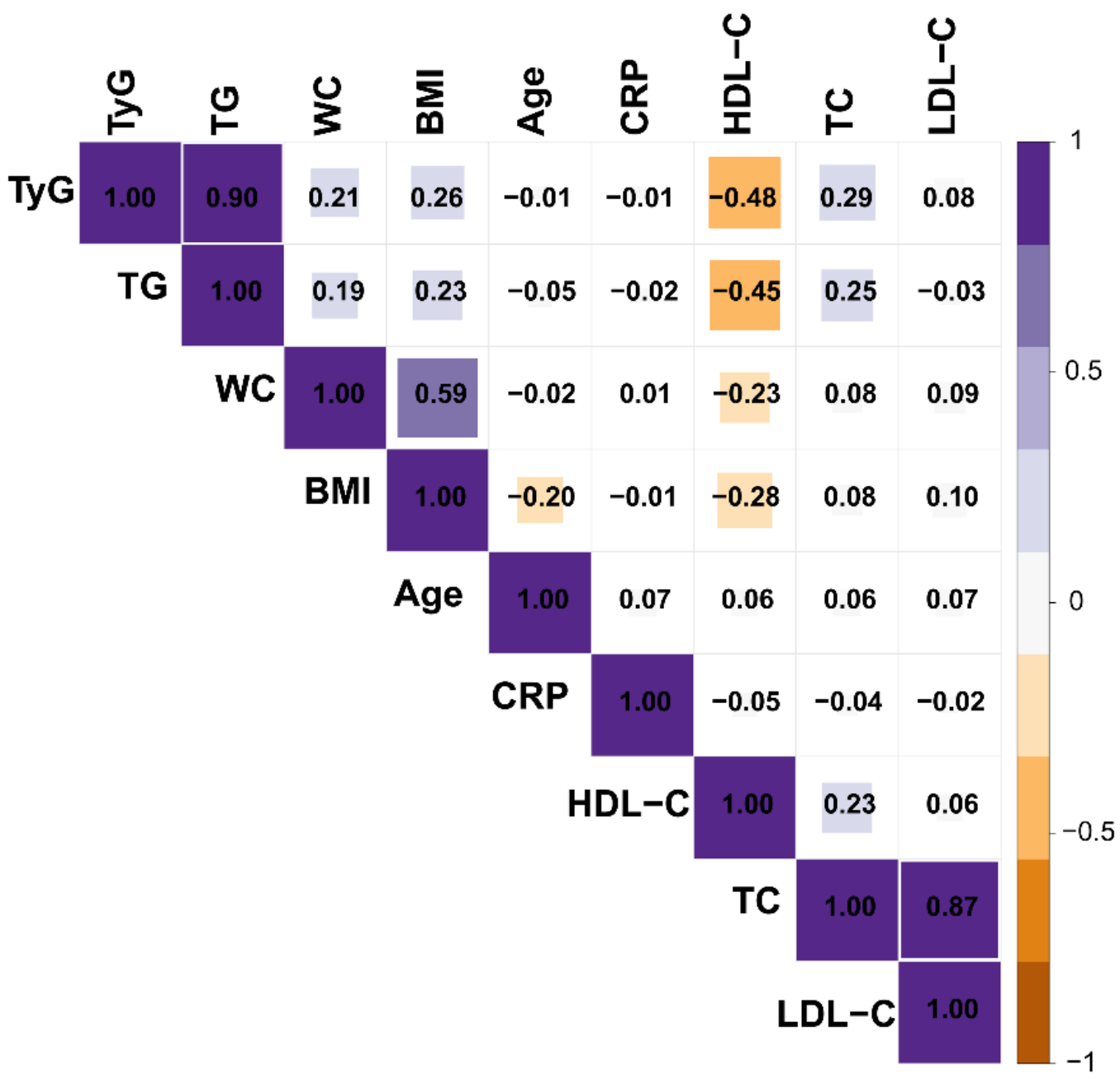

Figure S4. Heatmap of correlation matrix among independent variables.

Abbreviations: BMI body mass index, WC waist circumference, TC total Cholesterol, TG triglycerides, LDL-C low-density lipoprotein cholesterol, HDL-C high-density lipoprotein cholesterol, CRP C-reactive protein, TyG triglyceride-glucose.

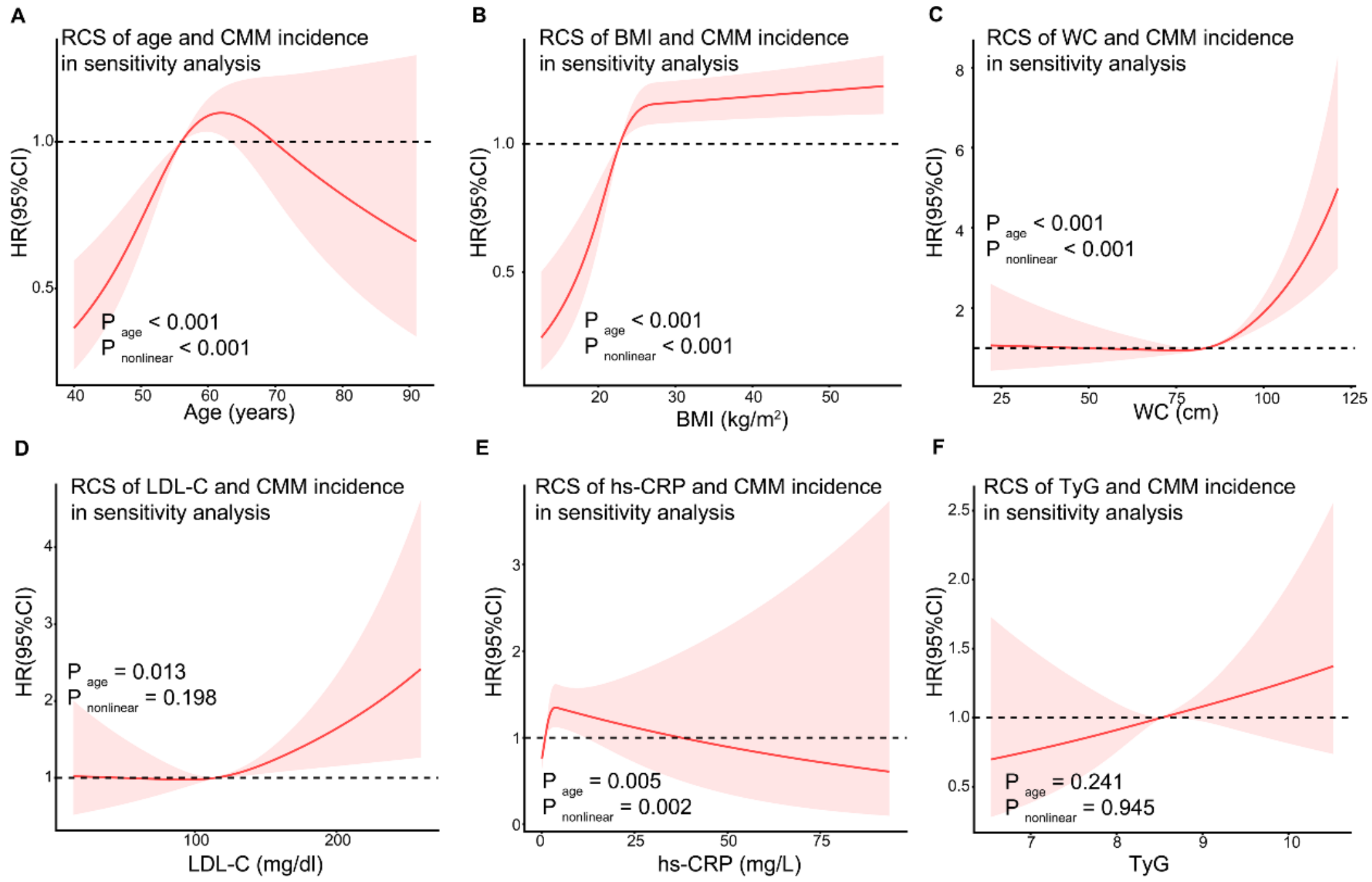

Figure S5. Sensitivity analysis: dose-response relationships between baseline metabolic parameters and incident CMM in the multiply imputed dataset (N = 11,846)

RCS analyses with three knots were performed using Cox proportional hazards regression models on the multiply imputed dataset (N = 11,846). All models were adjusted for sex, SBP, HbA1c, smoking, drinking, and the metabolic parameters (age, WC, LDL-C, hs-CRP, and TyG) not tested as the exposure in each panel. Solid lines represent the estimated hazard ratios (HRs), and shaded areas indicate the 95% confidence intervals. The panels show the dose-response curves for: **(A)** age, **(B)** BMI, **(C)** WC, **(D)** LDL-C, **(E)** hs-CRP, and **(F)** TyG.

Abbreviations: BMI, body mass index; CMM, cardiometabolic multimorbidity; CRP, C-reactive protein; hs-CRP, high-sensitivity C-reactive protein; HR, hazard ratio; LDL-C, low-density lipoprotein cholesterol; RCS, restricted cubic spline; SBP, systolic blood pressure; TyG, triglyceride-glucose index; WC, waist circumference.

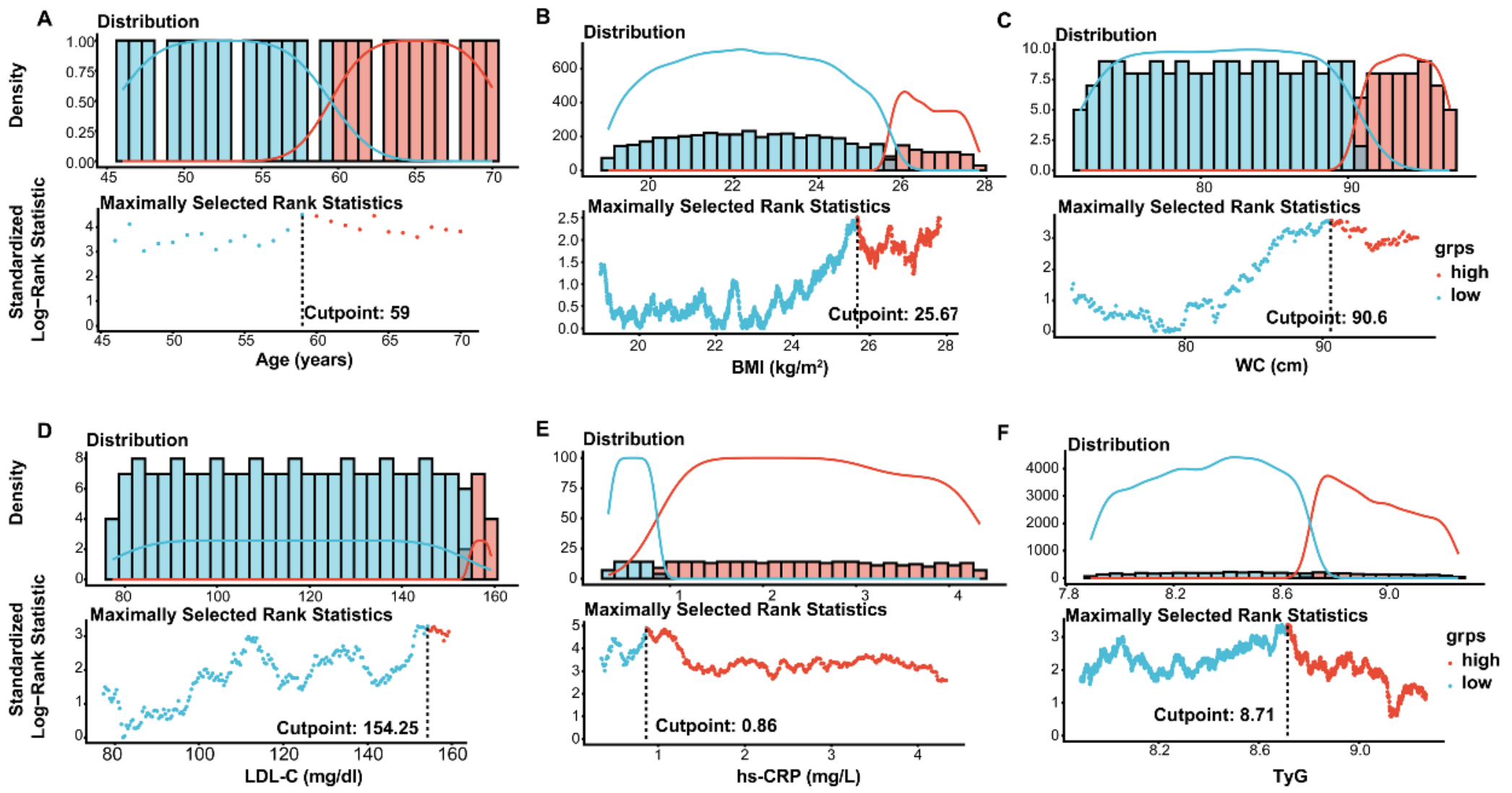

Figure S6. Determination of the optimal cut-off value for continuous variables using maximally selected rank statistics for CMM incidence risk.

The optimal cut-off point for each variable was identified by maximizing the standardized log-rank statistic over all possible cut-off values. The dashed vertical lines indicate the optimal cut-off points. The identified cut-off values were: (**A**) age, 60 years; (**B**) BMI, 25.6 kg/m²; (**C**) WC, 90.6 cm; (**D**) LDL-C, 154.3 mg/dL; (**E**) CRP, 0.86 mg/L; and (**F**) TyG index, 8.70.

Abbreviations: BMI body mass index, WC waist circumference, LDL-C low-density lipoprotein cholesterol, CRP C-reactive protein, TyG triglyceride-glucose, CMM cardiometabolic multimorbidity.

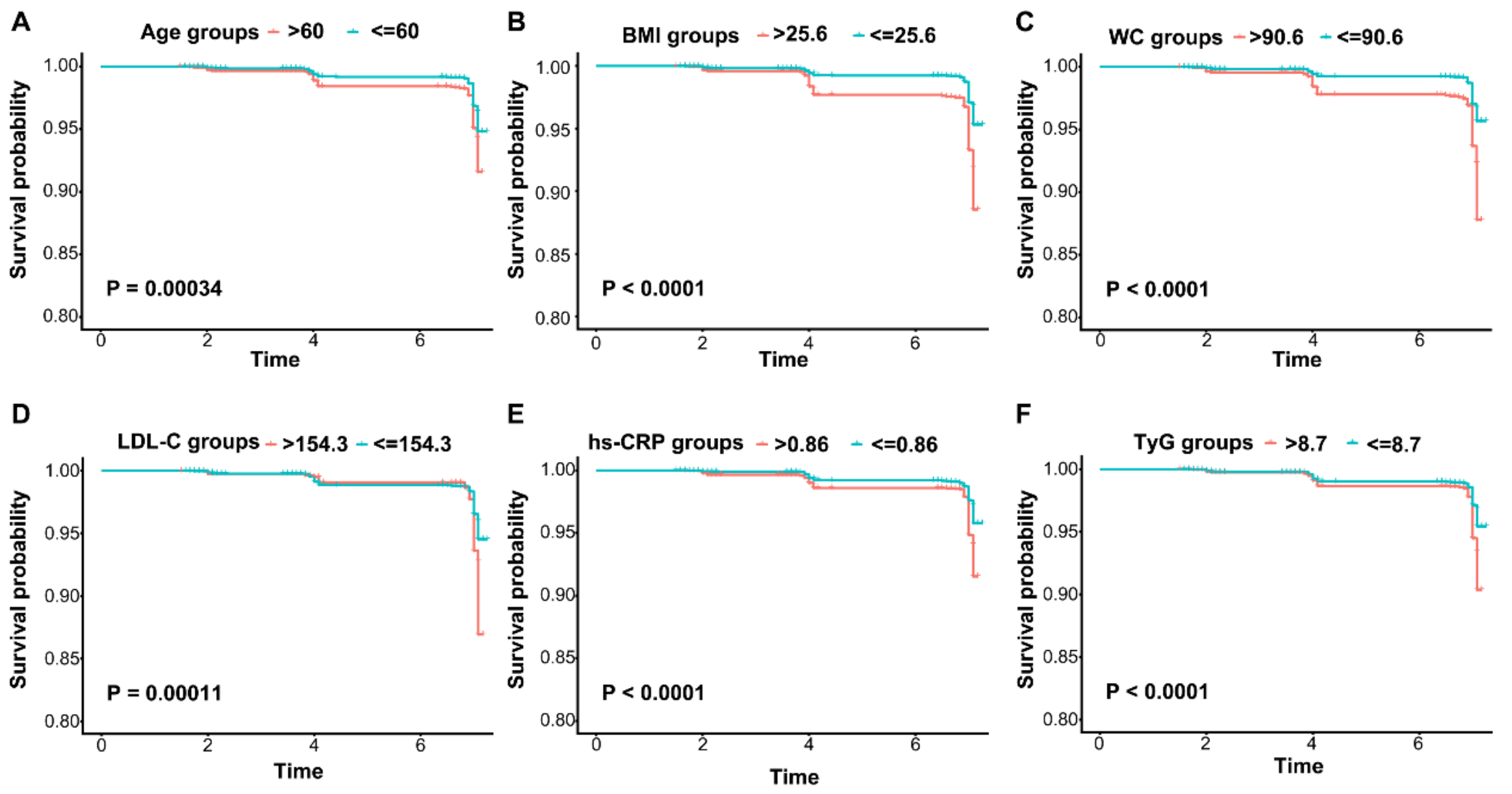

Figure S7. Kaplan-Meier curves for incident CMM stratified by optimal cut-off values derived from maximally selected rank statistics.

Participants were dichotomized according to the identified optimal cut-off points: (**A**) age (>60 *vs*. ≤60 years), (**B**) BMI (>25.6 *vs.* ≤25.6 kg/m²), (**C**) WC (>90.6 *vs.* ≤90.6 cm), (**D**) LDL-C (>154.3 *vs.* ≤154.3 mg/dL), (**E**) CRP (>0.86 *vs.* ≤0.86 mg/L), and (**F**) TyG (>8.70 *vs.* ≤8.70). P values were calculated using the log-rank test.

Abbreviations: BMI body mass index, WC waist circumference, LDL-C low-density lipoprotein cholesterol, CRP C-reactive protein, TyG triglyceride-glucose, CMM cardiometabolic multimorbidity.

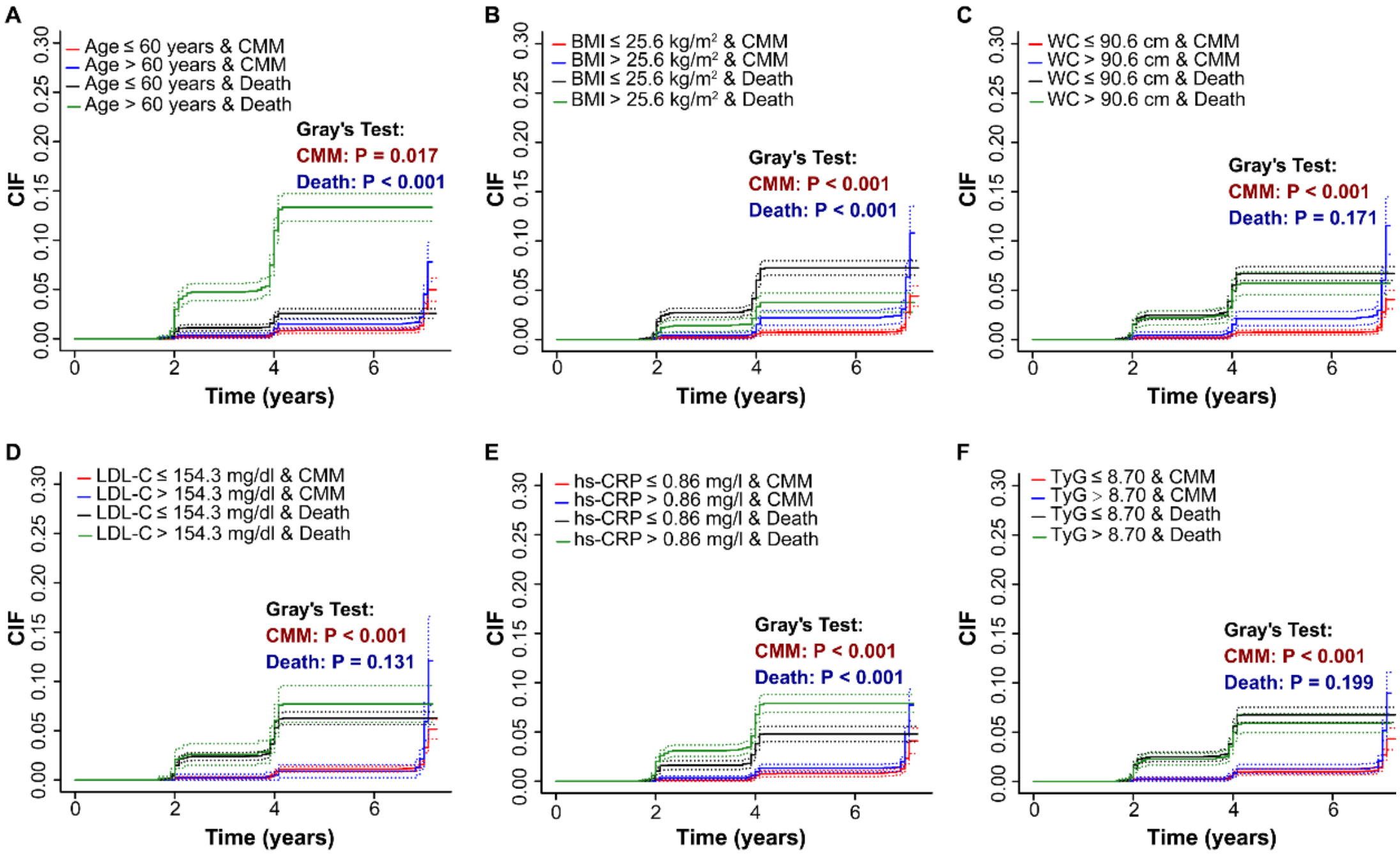

Figure S8. Cumulative incidence function (CIF) curves for incident CMM estimated using the Fine-Gray competing risks model.

Stratified according to the optimal cut-off values of: (**A**) age (60 years), (**B**) BMI (25.6 kg/m²), (**C**) WC (90.6 cm), (**D**) LDL-C (154.3 mg/dL), (**E**) CRP (0.86 mg/L), and (**F**) TyG (8.70). Death was treated as the competing event. P values were derived from Gray's test.

Abbreviations: BMI body mass index, WC waist circumference, LDL-C low-density lipoprotein cholesterol, CRP C-reactive protein, TyG triglyceride-glucose, CMM cardiometabolic multimorbidity.

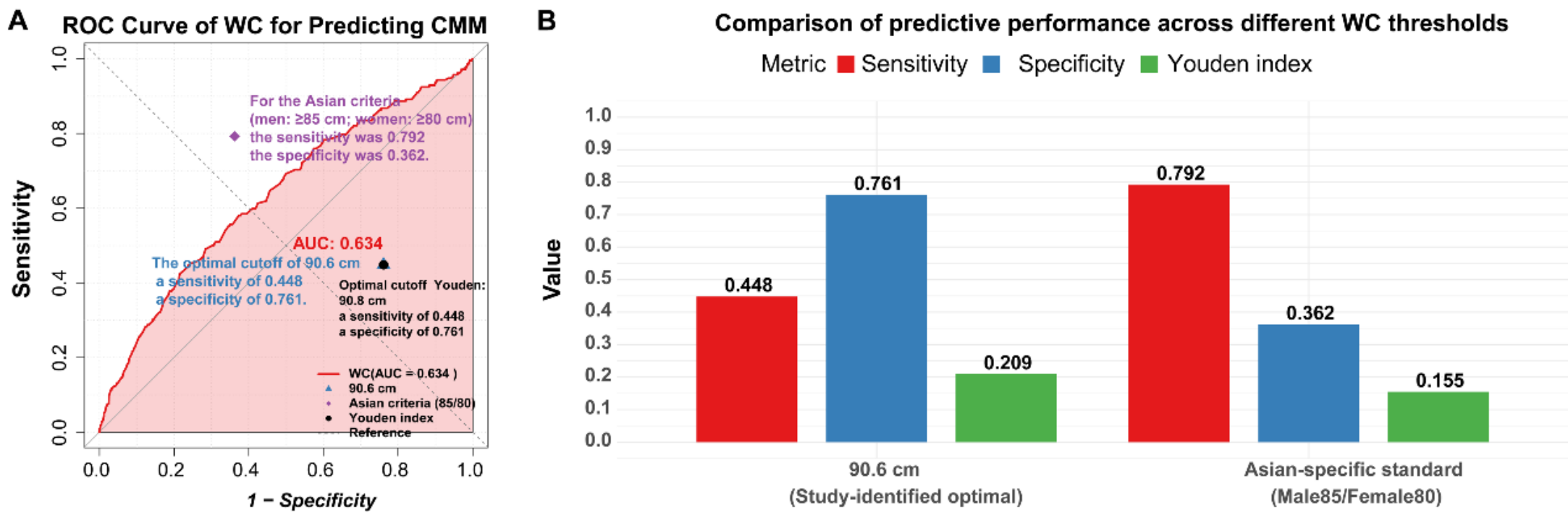

Figure S9. ROC curve of WC for predicting CMM and comparison of predictive performance across different WC thresholds

(A) ROC curve of WC for predicting CMM, with the study-optimal cutoff (90.6 cm) and Asian standard cutoff (85/80 cm for men/women) indicated. (B) Comparison of sensitivity, specificity, and Youden index between the two cutoffs. The study-optimal cutoff showed higher specificity and Youden index, while the Asian standard cutoff demonstrated higher sensitivity.

Abbreviations: WC, waist circumference; CMM, cardiometabolic multimorbidity; ROC, receiver operating characteristic; AUC, area under the curve; Youden index = sensitivity+specificity-1.

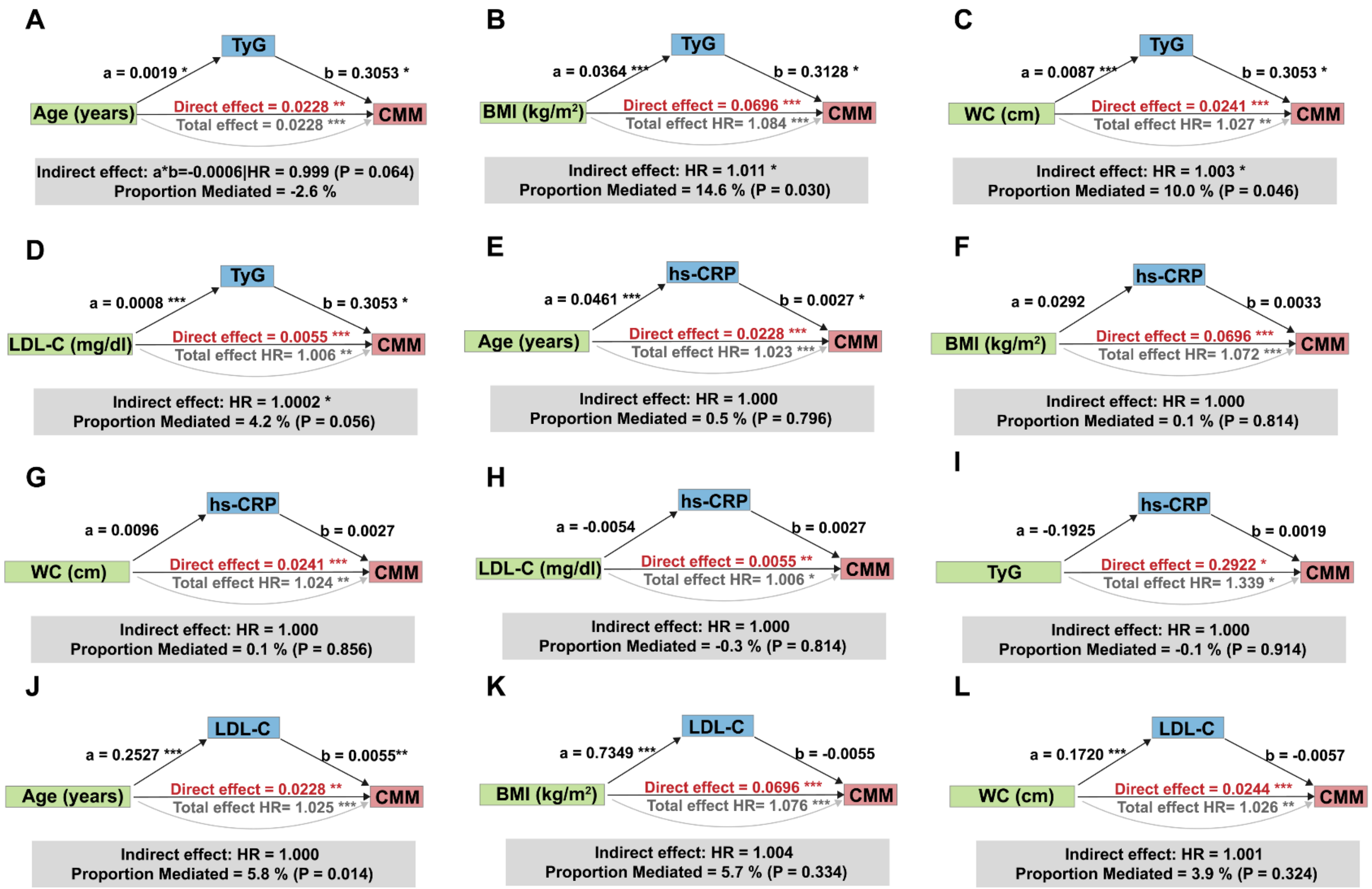

Figure S10. Path diagrams of the mediation analysis illustrating the direct and indirect effects of metabolic risk factors on CMM.

The analysis was performed using a regression-based counterfactual framework. All models estimated the natural direct effect (NDE), natural indirect effect (NIE), and total effect (TE) with adjustment for sex, SBP, and the metabolic covariates (including age, WC, LDL-C, hs-CRP, and TyG index) not tested as the exposure or mediator in each panel. Point estimates and 95% confidence intervals were derived from 1,000 bootstrap resamples using the percentile method. (**A–D**) Mediating role of the TyG index in the associations of age, BMI, WC, and LDL-C with incident CMM. (**E–I**) Mediating role of hs-CRP in the associations of age, BMI, WC, LDL-C, and TyG index with incident CMM. (**J–L**) Mediating role of LDL-C in the associations of age, BMI, and WC with incident CMM.

Abbreviations: BMI, body mass index; CMM, cardiometabolic multimorbidity; HDL-C, high-density lipoprotein cholesterol; hs-CRP, high-sensitivity C-reactive protein; LDL-C, low-density lipoprotein cholesterol; NDE, natural direct effect; NIE, natural indirect effect; SBP, systolic blood pressure; TE, total effect; TyG, triglyceride-glucose index; WC, waist circumference; NDE, natural direct effect; NIE, natural indirect effect; TE, total effect.

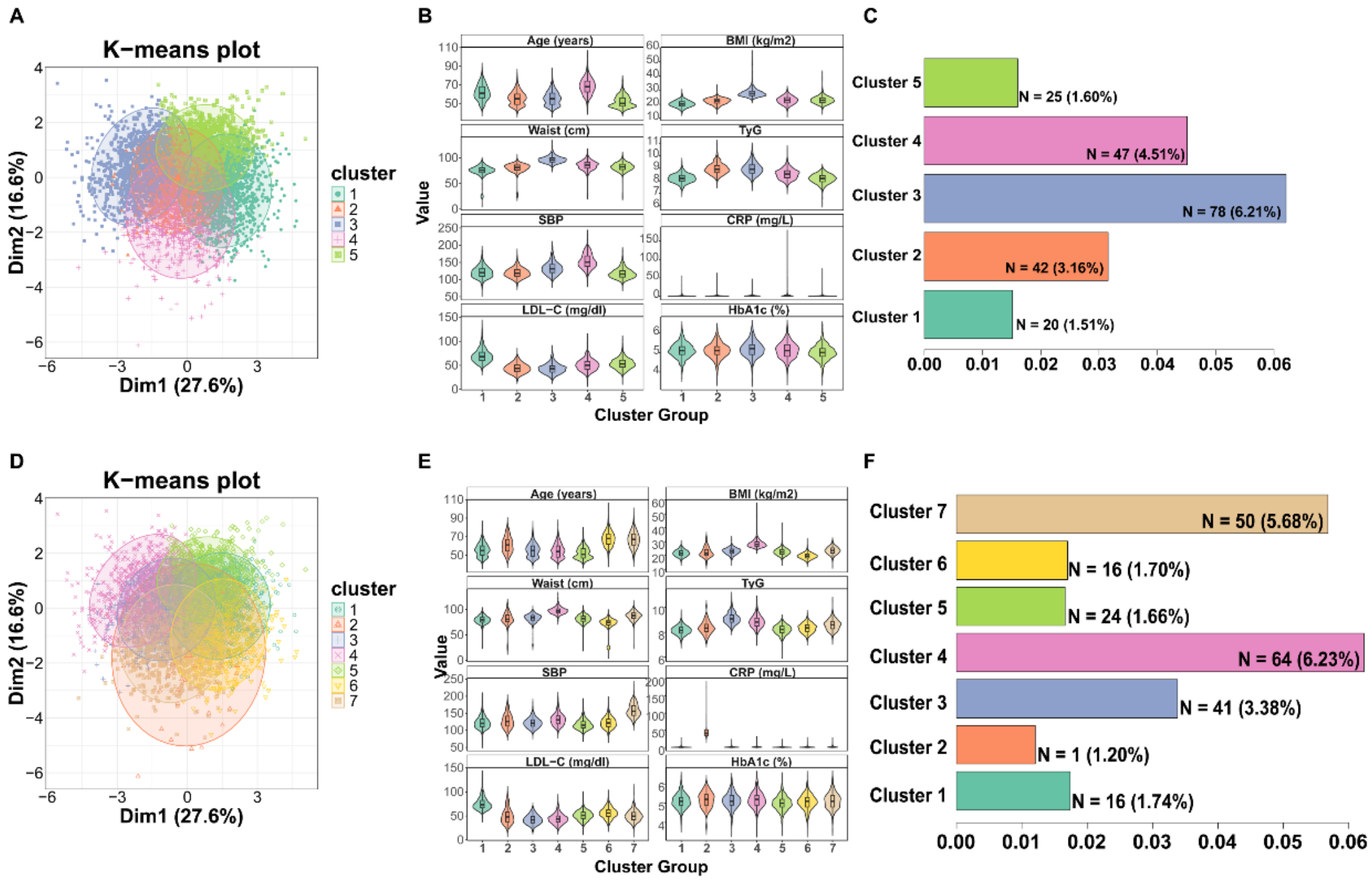

Figure S11. Identification of subgroups by K-means clustering and their association with incident CMM.

Two-dimensional visualization of participants classified into five (**A**) and seven (**D**) distinct clusters based on eight standardized cardiometabolic variables: age, BMI, WC, hs-CRP, TyG, SBP, HDL-C, and FPG. (**B**) Violin plots comparing the levels of individual metabolic indicators across the five clusters. (**E**) Violin plots comparing the levels of individual metabolic indicators across the seven clusters. **(C)** Bar plot showing the number and proportion of incident CMM events within five cluster. **(F)** Bar plot showing the number and proportion of incident CMM events within seven cluster. P value was calculated using the chi-square test.

Abbreviations: CMM cardiometabolic multimorbidity, BMI body mass index, WC waist circumference, HDL-C high-density lipoprotein cholesterol, hs-CRP high-sensitivity C-reactive protein, LDL-C low-density lipoprotein cholesterol, SBP systolic blood pressure, TyG triglyceride-glucose; FPG fasting plasma glucose.

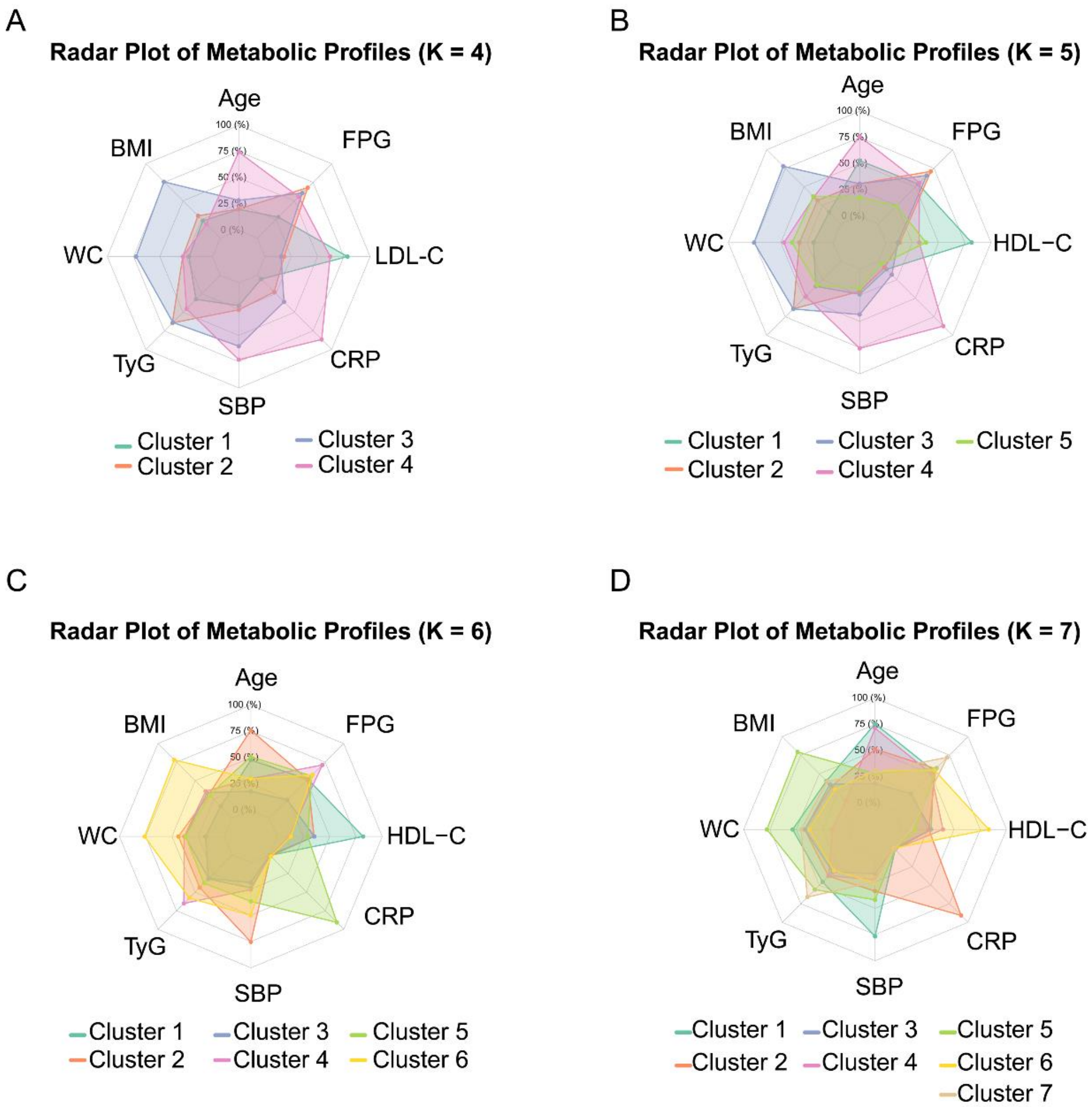

Figure S12. Radar plots of metabolic profiles across K-means clustering solutions (K = 4 to 7)

Radar plots showing the mean values of eight standardized cardiometabolic variables (age, BMI, WC, hs-CRP, TyG, SBP, HDL-C, and FPG) across (**A**) four, (**B**) five, (**C**) six, and (**D**) seven clusters. Each axis represents one variable, and each polygon represents a cluster. Clusters with elevated BMI, WC, and TyG consistently emerged across all solutions, supporting the stability of the obese–insulin-resistant phenotype. **Abbreviations:** BMI, body mass index; FPG, fasting plasma glucose; HDL-C, high-density lipoprotein cholesterol; hs-CRP, high-sensitivity C-reactive protein; SBP, systolic blood pressure; TyG, triglyceride-glucose index; WC, waist circumference.

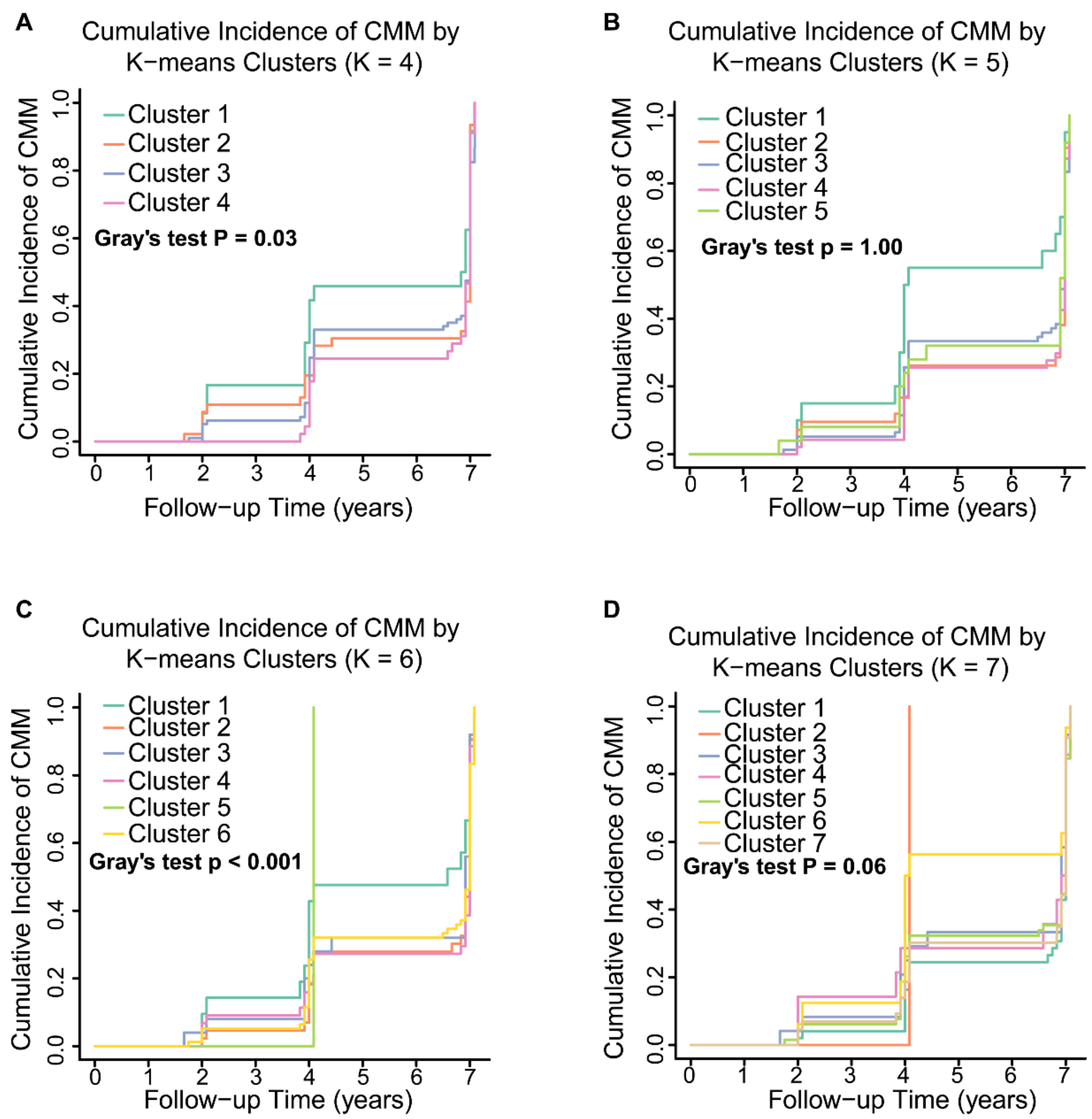

Figure S13. Cumulative incidence of CMM by K-means clusters across different clustering solutions

Cumulative incidence function (CIF) curves for incident cardiometabolic multimorbidity (CMM) stratified by (**A**) four, (**B**) five, (**C**) six, and (**D**) seven K-means clusters. All curves were estimated using the Fine-Gray competing risks model with all-cause death treated as the competing event. Gray's test p values are displayed in each panel.

Abbreviations: CIF, cumulative incidence function; CMM, cardiometabolic multimorbidity.

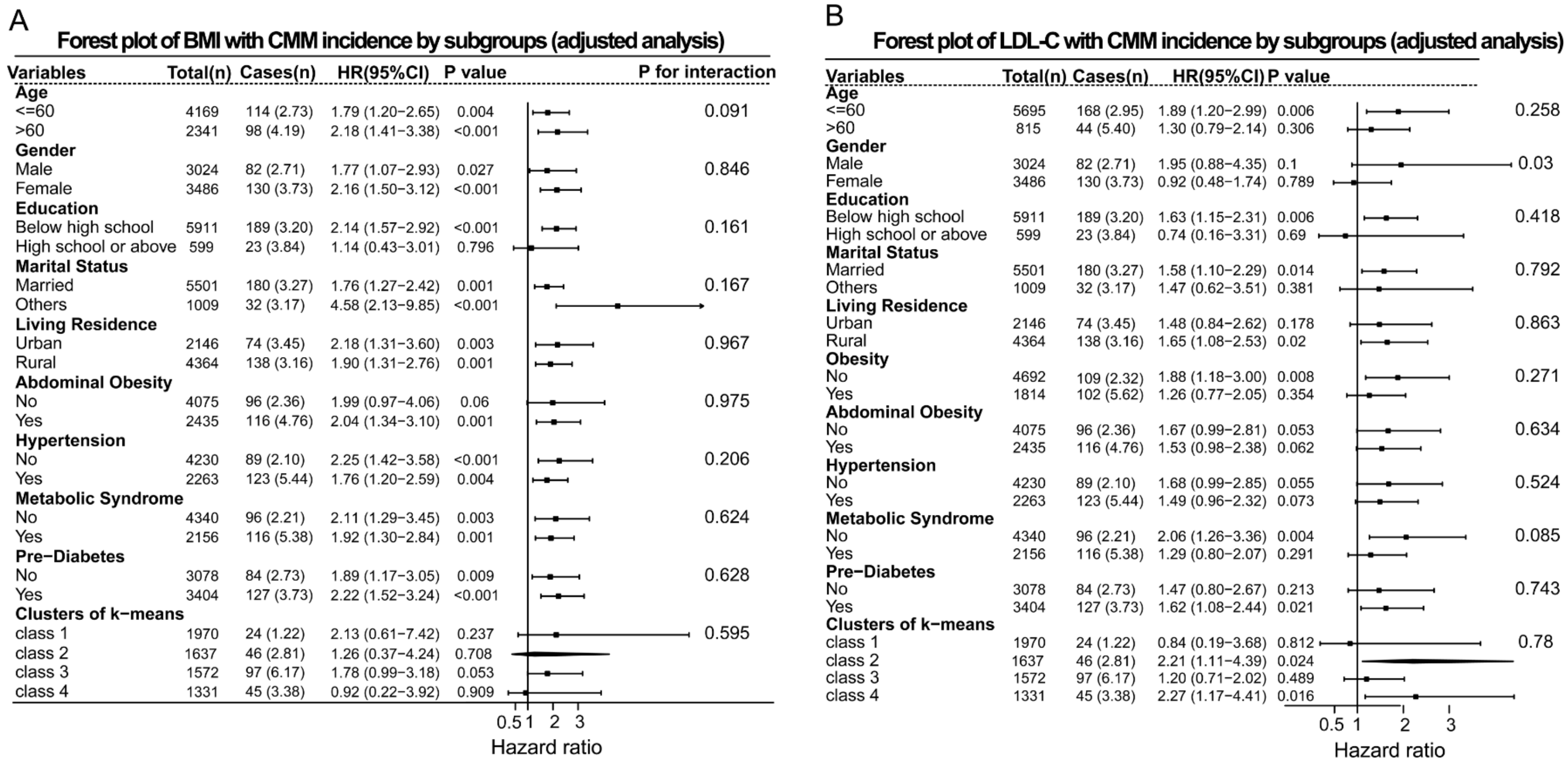

Figure S14. Forest plot of the association of BMI and LDL-C with incident CMM stratified by subgroups.

Hazard ratios (HRs) and 95% confidence intervals (CIs) were estimated using Cox proportional hazards regression models. All models were adjusted for sex, age, smoking status, drinking status, SBP, HbA1c, LDL-C, CRP, WC, and TyG index (excluding the variable used for stratification and exposure variable). Squares represent point estimates of HRs, and horizontal lines represent 95% CIs. The dashed vertical line indicates HR = 1 (null effect). P values for interaction were calculated to assess potential effect modification. (**A**) Forest plot of BMI (≤ 25.6

vs. >25.6 kg/m²) with CMM incidence by subgroups; (**B**) Forest plot of LDL-C (≤ 154.3 *vs.* >154.3 mg/dL) with CMM incidence by subgroups.

Abbreviations: CMM, cardiometabolic multimorbidity; BMI, body mass index; WC, waist circumference; CI, confidence interval; CRP, C-reactive protein; HbA1c, glycated hemoglobin; HR, hazard ratio; LDL-C, low-density lipoprotein cholesterol; SBP, systolic blood pressure; TyG, triglyceride-glucose.

Table S1. Baseline characteristics of included and excluded participants

Variables	Included (N=6510)	Excluded (N=11198)	P value	SMD
Age, mean (SD)	57.96 (9.41)	58.81 (10.58)	<0.001	0.085
Sex				
Male	3024 (46.5)	5454 (48.7)	0.004	0.045
Female	3486 (53.5)	5742 (51.3)		
Smoking				
Never	3985 (61.4)	6634 (63.7)	<0.001	0.098
Former	479 (7.4)	938 (9.0)		
Current	2030 (31.3)	2841 (27.3)		
Drinking				
Never	3815 (58.6)	6518 (59.1)	0.001	0.057
Former	480 (7.4)	963 (8.7)		
Current	2212 (34.0)	3555 (32.2)		
Obesity				
No	4692 (72.1)	4731 (66.5)	<0.001	0.122
Yes	1814 (27.9)	2384 (33.5)		
Central obesity				
No	4075 (62.6)	4027 (55.5)	<0.001	0.145
Yes	2435 (37.4)	3232 (44.5)		
SBP, mean (SD)	127.61 (20.99)	131.36 (21.91)	<0.001	0.175
DBP, mean (SD)	74.65 (12.19)	76.24 (12.26)	<0.001	0.130
WC, mean (SD)	83.33 (12.03)	85.12 (13.08)	<0.001	0.142
BMI, mean (SD)	23.21 (3.67)	24.75 (48.03)	0.01	0.045
FGP, mean (SD)	99.87 (11.66)	123.55 (51.75)	<0.001	0.631
HbA1c, mean (SD)	5.09 (0.40)	5.47 (1.11)	<0.001	0.452
TC, mean (SD)	191.98 (37.09)	194.21 (41.03)	0.002	0.057
TG, mean (SD)	119.34 (73.93)	154.60 (141.18)	<0.001	0.313
HDL-C, mean (SD)	52.45 (15.02)	48.81 (15.48)	<0.001	0.239
LDL-C, mean (SD)	116.64 (33.70)	115.18 (36.37)	0.025	0.042
Hs-CRP, mean (SD)	2.43 (6.94)	3.26 (8.40)	<0.001	0.107
TyG, mean (SD)	8.54 (0.55)	8.89 (0.77)	<0.001	0.516

Data are presented as mean (SD) for continuous variables and n (%) for categorical variables. P values were derived from t-tests or Wilcoxon rank-sum tests for continuous variables and chi-squared tests or Fisher's exact tests for categorical variables, as appropriate. SMD, standardized mean difference, was calculated to quantify the magnitude of between-group differences independent of sample size. SMD < 0.1 indicates negligible difference; 0.1–0.2 indicates small difference; 0.2–0.3 indicates moderate difference; > 0.3 indicates substantial difference. The Excluded group comprised participants removed for baseline prevalent CMM components (stroke, diabetes, or heart disease), missing CMM outcome data, or anomalous follow-up records. Expectedly, the Excluded group exhibited a less

favorable metabolic profile, particularly in glycemic and lipid parameters (FPG SMD = 0.631; HbA1c SMD = 0.452; TG SMD = 0.313), consistent with the exclusion of participants with baseline diabetes. Key anthropometric and demographic variables were well balanced between groups (age, sex, BMI, LDL-C all SMD < 0.1).

Abbreviations: SBP, systolic blood pressure; DBP, diastolic blood pressure; WC, waist circumference; BMI, body mass index; FPG, fasting plasma glucose; HbA1c, glycated hemoglobin; TC, total cholesterol; TG, triglycerides; HDL-C, high-density lipoprotein cholesterol; LDL-C, low-density lipoprotein cholesterol; Hs-CRP, high-sensitivity C-reactive protein; TyG, triglyceride-glucose index; SMD, standardized mean difference.

Table S2. Variance inflation factors (VIF) across models with metabolic indicators

Variables	Age group		BMI group		WC group		LDL-C group		Hs-CRP group		TyG group	
	GVIF	GVIF^(1/(2*Df)	GVIF	GVIF^(1/(2*Df)	GVIF	GVIF^(1/(2*Df)	GVIF	GVIF^(1/(2*Df)	GVIF	GVIF^(1/(2*Df)	GVIF	GVIF^(1/(2*Df)
Age	-	-	1.161	1.078	1.116	1.056	1.119	1.058	1.117	1.057	1.116	1.057
Sex	2.366	1.538	2.360	1.536	2.342	1.530	2.333	1.527	2.353	1.534	2.348	1.532
Smoking	2.181	1.215	2.192	1.267	2.154	1.212	2.169	1.214	2.174	1.214	2.169	1.214
Drinking	1.476	1.102	1.460	1.099	1.458	1.099	1.462	1.100	1.468	1.001	1.462	1.100
SBP	1.119	1.058	1.138	1.067	1.144	1.069	1.154	1.074	1.153	1.074	1.154	1.074
HbA1c	1.050	1.025	1.046	1.023	1.046	1.023	1.041	1.020	1.047	1.023	1.044	1.022
LDL-C	1.048	1.024	1.046	1.023	1.049	1.024	-	-	1.052	1.026	1.052	1.026
WC	1.144	1.070	-	-	-	-	1.146	1.071	1.177	1.085	1.123	1.060
Hs-CRP	1.014	1.007	1.010	1.005	1.012	1.006	1.012	1.006	-	-	1.013	1.006
TyG	1.103	1.050	1.096	1.046	1.107	1.052	1.107	1.052	1.112	1.055	-	-

The table presents the variance inflation factors for all covariates included in the Cox proportional hazards models, with different adiposity and metabolic indicators (age group, BMI group, WC group, LDL-C group, Hs-CRP group, and TyG group) serving as the primary exposure variable in each respective model. For binary variables with one degree of freedom (Df = 1), VIF equals GVIF. For categorical variables with two degrees of freedom (smoking status, drinking status; Df = 2), the adjusted VIF (GVIF^(1/(2×Df))) is reported. "-" denotes that the variable served as the primary exposure in that model and was therefore not included as a covariate.

Abbreviations: VIF, variance inflation factor; GVIF, generalized variance inflation factor; Df, degrees of freedom; BMI, body mass index; WC, waist circumference; LDL-C, low-density lipoprotein cholesterol; Hs-CRP, high-sensitivity C-reactive protein; TyG, triglyceride-glucose index; SBP, systolic blood pressure; HbA1c, glycated hemoglobin.

Table S3. Sensitivity analysis of the association between variables and incident CMM using the Fine-Gray competing risks model.

Variables	Model 1		Model 2	
	SHR (95%CI)	P value	SHR (95%CI)	P value
Age				
≤60	1.00 (Reference)	-	1.00 (Reference)	-
>60	1.53 (1.17-2.00)	0.002	1.34 (1.01-1.78) [a]	0.042
BMI				
≤ 25.6	1.00 (Reference)	-	1.00 (Reference)	-
> 25.6	2.44 (1.86-3.19)	<0.001	2.03 (1.51-2.72) [b]	<0.001
WC				
≤ 90.6	1.00 (Reference)	-	1.00 (Reference)	-
> 90.6	2.45 (1.87-3.20)	<0.001	1.92 (1.43-2.56) [c]	<0.001
LDL-C				
≤ 154.3	1.00 (Reference)	-	1.00 (Reference)	-
> 154.3	1.87 (1.35-2.60)	<0.001	1.51 (1.08-2.11) [d]	0.02
CRP				
≤ 0.86	1.00 (Reference)	-	1.00 (Reference)	-
> 0.86	1.94 (1.45-2.58)	<0.001	1.49 (1.09-2.02) [e]	0.011
TyG				
≤ 8.7	1.00 (Reference)	-	1.00 (Reference)	-
> 8.7	1.90 (1.46-2.49)	<0.001	1.49 (1.12-1.98) [f]	0.007

SHR, subdistribution hazard ratio; **CI,** confidence interval. All models were estimated using the Fine-Gray competing risks model with death treated as the competing event.

Model 1: Unadjusted for any covariates.

Model 2: Adjusted for sex, smoking, drinking, SBP, and metabolic variables (age, WC, LDL-C, hs-CRP, TyG) excluding the exposure variable in each panel.

a Adjusted for sex, smoking, drinking, WC, SBP, HbA1c, LDL-C, hs-CRP, and TyG;

b Adjusted for sex, age, smoking, drinking, SBP, HbA1c, LDL-C, hs-CRP, and TyG;

c Adjusted for sex, age, smoking, drinking, SBP, HbA1c, LDL-C, hs-CRP, and TyG;

d Adjusted for sex, age, smoking, drinking, WC, SBP, HbA1c, hs-CRP, and TyG;

e Adjusted for sex, age, smoking, drinking, WC, SBP, HbA1c, LDL-C, and TyG;

f Adjusted for sex, age, smoking, drinking, WC, SBP, HbA1c, LDL-C, and hs-CRP.

All models were estimated using the Fine-Gray competing risks model with all-cause death as the competing event.

Abbreviations: CMM cardiometabolic multimorbidity, BMI body mass index, WC waist circumference, HDL-C high-density lipoprotein cholesterol, hs-CRP high-sensitivity C-reactive protein, LDL-C low-density lipoprotein cholesterol, SBP systolic blood pressure, TyG triglyceride-glucose.

Table S4. Comparison of predictive performance between the study-identified optimal cutoff and the Asian standard for WC in predicting CMM

Threshold	Cutoff	TP	TN	FP	FN	Sensitivity	Specificity	Accuracy	PPV	NPV	Youden	LR_plus	LR_minus
Stufy optimal	90.6	95	4793	1505	117	0.448	0.761	0.751	0.059	0.976	0.209	1.88	0.73
Asian standard	Male85/ Female80	168	2281	4017	44	0.793	0.362	0.376	0.040	0.981	0.155	1.24	0.57

Abbreviations: TP, true positives; TN, true negatives; FP, false positives; FN, false negatives; PPV, positive predictive value; NPV, negative predictive value; LR_plus, positive likelihood ratio; LR_minus, negative likelihood ratio.

Data are presented as raw counts or performance metrics. The study-optimal cutoff of 90.6 cm was derived from the Youden index. The Asian standard followed the recommended clinical diagnostic criteria for Asian populations. Sensitivity, specificity, accuracy, PPV, NPV, Youden index, and likelihood ratios were calculated accordingly.

Table S5. Average silhouette width across K-means clustering solutions (K = 2 to 10)

K	Average Silhouette Width
2	0.1845
3	0.1942
4	0.1563
5	0.1452
6	0.1433
7	0.1469
8	0.1339
9	0.1377
10	0.1290

Note: Average silhouette width was calculated using the silhouette () function from the cluster package in R. Values below 0.25 indicate modest cluster separation, which is consistent with the continuous nature of cardiometabolic risk profiles in population-based data.

Abbreviation: K, number of clusters.

Table S6. Within-cluster sum of squares and variance explained across K-means clustering solutions (K = 2 to 10)

K	Total WCSS	Variance Explained	WCSS Reduction
2	42692.80	18.01	-
3	38711.08	25.66	9.33
4	35009..89	32.77	9.56
5	32555.87	37.48	7.01
6	30537.87	41.36	6.20
7	28714.22	44.86	5.97
8	27287.15	47.60	4.97
9	26051.64	49.97	4.53
10	25000.61	51.99	4.03

K-means clustering was performed with 50 random initializations (nstart = 50, iter.max = 100) using eight standardized cardiometabolic variables (age, BMI, WC, hs-CRP, TyG, SBP, HDL-C, and FPG). Total WCSS, total within-cluster sum of squares. Variance Explained, the proportion of total variance explained by the clustering solution, calculated as (TSS − WCSS) / TSS × 100%, where TSS is the total sum of squares. WCSS Reduction, the percentage reduction in WCSS relative to the solution with K − 1, calculated as (WCSS_K − WCSS_{K+1}) / WCSS_K × 100%. The diminishing WCSS reduction beyond K = 4 (from 9.56% to 7.01%) marks the primary elbow point, while the consistently modest reductions through K = 6 (6.20%) and the drop below 5% at K = 8 (4.97%) provide quantitative support for selecting K = 4 as a parsimonious solution and K = 6 as capturing additional metabolic heterogeneity without excessive fragmentation.

Abbreviations: TSS, total sum of squares; WCSS, within-cluster sum of squares; K, number of clusters.

Table S7. Stability of K-means clustering solutions (K = 4 to 7) assessed by the adjusted Rand index across 20 repeated runs

K	Mean ARI	Median ARI	Min ARI	Max ARI	Comparisons (N)
4	0.9594	0.9891	0.8373	1.0000	190
5	0.9661	0.9753	0.8802	1.0000	190
6	0.9843	0.9854	0.9672	1.0000	190
7	0.8480	0.9209	0.3615	1.0000	190

K-means clustering was performed with 50 random initializations (nstart = 50, iter.max = 100) for each of the 20 independent runs. The adjusted Rand index (ARI) was calculated for each pair of runs (C (20,2) = 190 pairwise comparisons). ARI = 1 indicates perfect agreement; ARI ≈ 0 indicates agreement no better than chance. Mean ARI values > 0.90 for K = 4, 5, and 6 indicate high stability, while the lower mean ARI for K = 7 (0.85) and wider range (0.36–1.00) suggest reduced reproducibility, consistent with over-fragmentation at higher K values.

Abbreviations: ARI, adjusted Rand index; K, number of clusters.

Table S8. Sensitivity analysis: associations of baseline metabolic parameters with risk of incident CMM after excluding events occurring within the first 2 years of follow-up (2-year lag analysis).

Variables	Total N	Cases N (%)	Model 1		Model 2	
			HR (95%CI)	P values	HR (95%CI)	P values
Age						
≤ 60	4169	114 (2.73)	1.00	-	1.00	-
> 60	2339	96 (4.10)	1.67 (1.27-2.19)	<0.001	1.36 (1.03-1.81) [a]	0.032
BMI						
≤ 25.6	5000	120 (2.40)	1.00	-	1.00	-
> 25.6	1508	90 (5.97)	2.36 (1.80-3.11)	<0.001	1.99 (1.48-2.69) [b]	<0.001
WC						
≤ 90.6	4909	116 (2.36)	1.00	-	1.00	-
> 90.6	1599	94 (5.88)	2.43 (1.85-3.19)	<0.001	1.86 (1.40-2.49) [c]	<0.001
LDL-C						
≤ 154.3	5693	166 (2.92)	1.00	-	1.00	-
> 154.3	815	44 (5.40)	1.94 (1.39-2.70)	<0.001	1.58 (1.13-2.21) [d]	0.008
CRP						
≤ 0.86	3029	66 (2.18)	1.00	-	1.00	-
> 0.86	3479	144 (4.14)	2.02 (1.51-2.71)	<0.001	1.51 (1.11-2.05) [e]	0.008
TyG						
≤ 8.7	4145	101 (2.44)	1.00	-	1.00	-
> 8.7	2363	109 (4.61)	1.90 (1.45-2.49)	<0.001	1.43 (1.07-1.89) [f]	0.014

HR, hazard ratio; **CI**, confidence interval. All models were estimated using Cox proportional hazards regression. For the lag analysis, we excluded participants who developed CMM (n = 2) within the first 2 years of follow-up, retaining 6,508 participants with 210 CMM events. The Fine-Gray competing risks model was then re-estimated using these lag datasets.

Model 1: Unadjusted for any covariates.

Model 2: Adjusted for sex, smoking, drinking, SBP, HbA1c, and the listed metabolic variables (age, WC, LDL-C, hs-CRP, TyG) excluding the exposure variable in each panel.

a Adjusted for sex, smoking, drinking, WC, SBP, HbA1c, LDL-C, hs-CRP, and TyG;

b Adjusted for sex, age, smoking, drinking, SBP, HbA1c, LDL-C, hs-CRP, and TyG;

c Adjusted for sex, age, smoking, drinking, SBP, HbA1c, LDL-C, hs-CRP, and TyG;

d Adjusted for sex, age, smoking, drinking, WC, SBP, HbA1c, hs-CRP, and TyG;

e Adjusted for sex, age, smoking, drinking, WC, SBP, HbA1c, LDL-C, and TyG;

f Adjusted for sex, age, smoking, drinking, WC, SBP, HbA1c, LDL-C, and hs-CRP.

Abbreviations: CMM cardiometabolic multimorbidity, BMI body mass index, WC waist circumference, HDL-C high-density lipoprotein cholesterol, hs-CRP high-sensitivity C-reactive protein, LDL-C low-density lipoprotein cholesterol, SBP systolic blood pressure, TyG triglyceride-glucose.

Table S9. Sensitivity analysis: Fine-Gray competing risks model for the associations of baseline metabolic parameters with risk of incident CMM after excluding events occurring within the first 2 years of follow-up (2-year lag analysis).

Variables	Model 1		Model 2	
	SHR (95%CI)	P value	SHR (95%CI)	P value
Age				
≤60	1.00 (Reference)	-	1.00 (Reference)	-
>60	1.50 (1.15-1.96)	0.003	1.31 (0.99-1.75) [a]	0.061
BMI				
≤ 25.6	1.00 (Reference)	-	1.00 (Reference)	-
> 25.6	2.43 (1.85-3.19)	<0.001	2.00 (1.48-2.69) [b]	<0.001
WC				
≤ 90.6	1.00 (Reference)	-	1.00 (Reference)	-
> 90.6	2.44 (1.86-3.20)	<0.001	1.91 (1.42-2.56) [c]	<0.001
LDL-C				
≤ 154.3	1.00 (Reference)	-	1.00 (Reference)	-
> 154.3	1.90 (1.37-2.63)	<0.001	1.55 (1.11-2.16) [d]	0.011
CRP				
≤ 0.86	1.00 (Reference)	-	1.00 (Reference)	-
> 0.86	1.95 (1.46-2.61)	<0.001	1.48 (1.08-2.01) [e]	0.014
TyG				
≤ 8.7	1.00 (Reference)	-	1.00 (Reference)	-
> 8.7	1.90 (1.46-2.49)	<0.001	1.44 (1.08-1.91) [f]	0.013

SHR, subdistribution hazard ratio; **CI**, confidence interval. All models were estimated using the Fine-Gray competing risks model with death treated as the competing event. For the lag analysis, we excluded participants who developed CMM (n = 2) within the first 2 years of follow-up, retaining 6,508 participants with 210 CMM events. The Fine-Gray competing risks model was then re-estimated using these lag datasets.

Model 1: Unadjusted for any covariates.

Model 2: Adjusted for sex, smoking, drinking, SBP, HbA1c, and the listed metabolic variables (age, WC, LDL-C, hs-CRP, TyG) excluding the exposure variable in each panel.

a Adjusted for sex, smoking, drinking, WC, SBP, HbA1c, LDL-C, hs-CRP, and TyG;
b Adjusted for sex, age, smoking, drinking, SBP, HbA1c, LDL-C, hs-CRP, and TyG;
c Adjusted for sex, age, smoking, drinking, SBP, HbA1c, LDL-C, hs-CRP, and TyG;
d Adjusted for sex, age, smoking, drinking, WC, SBP, HbA1c, hs-CRP, and TyG;
e Adjusted for sex, age, smoking, drinking, WC, SBP, HbA1c, LDL-C, and TyG;
f Adjusted for sex, age, smoking, drinking, WC, SBP, HbA1c, LDL-C, and hs-CRP.

Abbreviations: CMM cardiometabolic multimorbidity, BMI body mass index, WC waist circumference, HDL-C high-density lipoprotein cholesterol, hs-CRP high-sensitivity C-reactive protein, LDL-C low-density lipoprotein cholesterol, SBP systolic blood pressure, TyG triglyceride-glucose.

Table S10. Sensitivity analysis: associations of baseline metabolic parameters with risk of incident CMM and death after excluding events occurring within the first 2 years of follow-up (2-year lag analysis).

Variables	Total N	Cases N (%)	Model 1		Model 2	
			HR (95%CI)	P values	HR (95%CI)	P values
Age						
≤ 60	4157	114 (2.74)	1.00	-	1.00	-
> 60	2322	96 (4.13)	1.67 (1.27-2.19)	<0.001	1.36 (1.03-1.81) [a]	0.032
BMI						
≤ 25.6	4974	120 (2.41)	1.00	-	1.00	-
> 25.6	1505	90 (5.98)	2.36 (1.80-3.11)	<0.001	1.99 (1.48-2.69) [b]	<0.001
WC						
≤ 90.6	4886	116 (2.37)	1.00	-	1.00	-
> 90.6	1593	94 (5.90)	2.43 (1.85-3.19)	<0.001	1.86 (1.40-2.49) [c]	<0.001
LDL-C						
≤ 154.3	5669	166 (2.93)	1.00	-	1.00	-
> 154.3	810	44 (5.43)	1.94 (1.39-2.70)	<0.001	1.58 (1.13-2.21) [d]	0.008
CRP						
≤ 0.86	3020	66 (2.19)	1.00	-	1.00	-
> 0.86	3459	144 (4.16)	2.02 (1.51-2.71)	<0.001	1.51 (1.11-2.05) [e]	0.008
TyG						
≤ 8.7	4127	101 (2.45)	1.00	-	1.00	-
> 8.7	2352	109 (4.63)	1.90 (1.45-2.49)	<0.001	1.43 (1.07-1.89) [f]	0.014

HR, hazard ratio; **CI**, confidence interval. All models were estimated using Cox proportional hazards regression. For the lag analysis, we excluded participants who developed CMM (n = 2) and died (n = 29) within the first 2 years of follow-up, retaining 6,479 participants with 210 CMM events.

Model 1: Unadjusted for any covariates.

Model 2: Adjusted for sex, smoking, drinking, SBP, HbA1c, and the listed metabolic variables (age, WC, LDL-C, hs-CRP, TyG) excluding the exposure variable in each panel.

a Adjusted for sex, smoking, drinking, WC, SBP, HbA1c, LDL-C, hs-CRP, and TyG;
b Adjusted for sex, age, smoking, drinking, SBP, HbA1c, LDL-C, hs-CRP, and TyG;
c Adjusted for sex, age, smoking, drinking, SBP, HbA1c, LDL-C, hs-CRP, and TyG;
d Adjusted for sex, age, smoking, drinking, WC, SBP, HbA1c, hs-CRP, and TyG;
e Adjusted for sex, age, smoking, drinking, WC, SBP, HbA1c, LDL-C, and TyG;
f Adjusted for sex, age, smoking, drinking, WC, SBP, HbA1c, LDL-C, and hs-CRP.

Abbreviations: CMM cardiometabolic multimorbidity, BMI body mass index, WC waist circumference, HDL-C high-density lipoprotein cholesterol, hs-CRP high-sensitivity C-reactive protein, LDL-C low-density lipoprotein cholesterol, SBP systolic blood pressure, TyG triglyceride-glucose.

Table S11. Sensitivity analysis: Fine-Gray competing risks model for the associations of baseline metabolic parameters with risk of incident CMM and death after excluding events occurring within the first 2 years of follow-up (2-year lag analysis).

Variables	Model 1		Model 2	
	SHR (95%CI)	P value	SHR (95%CI)	P value
Age				
≤60	1.00 (Reference)	-	1.00 (Reference)	-
>60	1.51 (1.15-1.97)	0.003	1.32 (0.99-1.75) [a]	0.058
BMI				
≤ 25.6	1.00 (Reference)	-	1.00 (Reference)	-
> 25.6	2.42 (1.85-3.18)	<0.001	1.98 (1.47-2.67) [b]	<0.001
WC				
≤ 90.6	1.00 (Reference)	-	1.00 (Reference)	-
> 90.6	2.44 (1.86-3.19)	<0.001	1.90 (1.41-2.54) [c]	<0.001
LDL-C				
≤ 154.3	1.00 (Reference)	-	1.00 (Reference)	-
> 154.3	1.90 (1.37-2.64)	<0.001	1.55 (1.11-2.16) [d]	0.011
CRP				
≤ 0.86	1.00 (Reference)	-	1.00 (Reference)	-
> 0.86	1.96 (1.46-2.62)	<0.001	1.48 (1.09-2.02) [e]	0.013
TyG				
≤ 8.7	1.00 (Reference)	-	1.00 (Reference)	-
> 8.7	1.90 (1.46-2.49)	<0.001	1.44 (1.08-1.92) [f]	0.013

SHR, subdistribution hazard ratio; **CI,** confidence interval. All models were estimated using the Fine-Gray competing risks model with death treated as the competing event. For the lag analysis, we excluded participants who developed CMM (n = 2) and died (n = 29) within the first 2 years of follow-up, retaining 6,479 participants with 210 CMM events.

Model 1: Unadjusted for any covariates.

Model 2: Adjusted for sex, smoking, drinking, SBP, HbA1c, and the listed metabolic variables (age, WC, LDL-C, hs-CRP, TyG) excluding the exposure variable in each panel.

a Adjusted for sex, smoking, drinking, WC, SBP, HbA1c, LDL-C, hs-CRP, and TyG;
b Adjusted for sex, age, smoking, drinking, SBP, HbA1c, LDL-C, hs-CRP, and TyG;
c Adjusted for sex, age, smoking, drinking, SBP, HbA1c, LDL-C, hs-CRP, and TyG;
d Adjusted for sex, age, smoking, drinking, WC, SBP, HbA1c, hs-CRP, and TyG;
e Adjusted for sex, age, smoking, drinking, WC, SBP, HbA1c, LDL-C, and TyG;
f Adjusted for sex, age, smoking, drinking, WC, SBP, HbA1c, LDL-C, and hs-CRP.

Abbreviations: CMM cardiometabolic multimorbidity, BMI body mass index, WC waist circumference, HDL-C high-density lipoprotein cholesterol, hs-CRP high-sensitivity C-reactive protein, LDL-C low-density lipoprotein cholesterol, SBP systolic blood pressure, TyG triglyceride-glucose.

Table S12. Comparison of baseline characteristics between the original dataset and the multiply imputed dataset.

Variables	Original dataset (N=11846)	Multiple imputation dataset(N=11846)	P value
Sex			
Male	5812 (49.0%)	5812 (49.1%)	1.000
Female	6034 (50.90%)	6034 (50.90%)	
Age, mean (SD)	57.6 (9.67)	57.6 (9.67)	1.000
Smoking			
Never	7094 (62.20%)	7223 (61.00%)	0.140
Former	798 (7.00%)	861 (7.27%)	
Current	3506 (30.8%)	3762 (31.80%)	
Drinking			
Never	6823 (57.70)	6831 (57.70%)	0.999
Former	843 (7.13%)	846 (7.14%)	
Current	4162 (35.20%)	4169 (35.20%)	
Obesity			
No	6789 (72.40%)	8559 (72.30%)	0.881
Yes	2594 (27.60%)	3287 (27.70%)	
Central obesity			
No	5946 (62.90%)	7485 (63.20%)	0.671
Yes	3508 (37.10%)	4361 (36.80)	
Hypertension			
No	6136 (61.90%)	7405 (62.50%)	0.376
Yes	3774 (38.10%)	4441 (37.50%)	
SBP, mean (SD)	128 (21.0)	129 (21.2)	0.006
WC, mean (SD)	83.4 (12.0)	83.4 (12.0)	0.949
BMI, mean (SD)	23.6 (20.2)	23.5 (18.0)	0.680
HbA1c,	5.09 (0.40)	5.09 (0.40)	0.957
TC,	191 (37.0)	192 (37.9)	0.814
TG,	120 (75.8)	118 (70.0)	0.076
HDL-C	52.2 (15.0)	52.3 (15.1)	0.786
LDL-C	116 (33.5)	117 (34.1)	0.268
CRP,	2.43 (6.82)	2.38 (6.70)	0.610
TyG	8.55 (0.55)	8.55 (0.54)	0.937

Data are presented as n (%) for categorical variables and mean (SD) for continuous variables. P values were calculated using the chi-square test for categorical variables and the t-test or Wilcoxon rank-sum test for continuous variables.

Abbreviations: SBP, systolic blood pressure; WC, waist circumference; BMI, body mass index; HbA1c, glycated hemoglobin; TC, total cholesterol; TG, triglycerides; HDL-C, high-density lipoprotein cholesterol; LDL-C, low-density lipoprotein cholesterol; CRP, C-reactive protein; TyG, triglyceride-glucose index.

Table S13. Sensitivity analysis of the association between variables and incident CMM using the cox regression model using the multiply imputed dataset.

Variables	Total N	Cases N (%)	Model 1		Model 2	
			HR (95%CI)	P values	HR (95%CI)	P values
Age						
≤ 60	7799	211 (2.71)	1.00	-	1.00	-
> 60	4047	159 (3.93)	1.63 (1.33-2.00)	<0.001	1.32 (1.07-1.63) [a]	0.011
BMI						
≤ 25.6	9147	223 (2.44)	1.00	-	1.00	-
> 25.6	2699	147 (5.45)	2.18 (1.77-2.68)	<0.001	1.88 (1.51-2.34) [b]	<0.001
WC						
≤ 90.6	8992	215 (2.39)	1.00	-	1.00	-
> 90.6	2854	155 (5.43)	2.22 (1.81-2.73)	<0.001	1.79 (1.45-2.23) [c]	<0.001
LDL-C						
≤ 154.3	10298	298 (2.89)	1.00	-	1.00	-
> 154.3	1548	72 (4.65)	1.65 (1.28-2.14)	<0.001	1.42 (1.10-1.84) [d]	0.008
CRP						
≤ 0.86	5525	129 (2.33)	1.00	-	1.00	-
> 0.86	6321	241 (3.81)	1.70 (1.37-2.11)	<0.001	1.41 (1.13-1.75) [e]	0.002
TyG						
≤ 8.7	7364	187 (2.54)	1.00	-	1.00	-
> 8.7	4482	183 (4.08)	1.62 (1.32-2.99)	<0.001	1.29 (1.04-1.59) [f]	0.019

HR, hazard ratio; **CI**, confidence interval. All models were estimated using Cox proportional hazards regression. For the lag analysis, we excluded participants who developed CMM (n = 2) within the first 2 years of follow-up, retaining 6,508 participants with 210 CMM events. The Fine-Gray competing risks model was then re-estimated using these lag datasets.

Model 1: Unadjusted for any covariates.

Model 2: Adjusted for sex, smoking, drinking, SBP, HbA1c, and the listed metabolic variables (age, WC, LDL-C, hs-CRP, TyG) excluding the exposure variable in each panel.

a Adjusted for sex, smoking, drinking, WC, SBP, HbA1c, LDL-C, hs-CRP, and TyG;
b Adjusted for sex, age, smoking, drinking, SBP, HbA1c, LDL-C, hs-CRP, and TyG;
c Adjusted for sex, age, smoking, drinking, SBP, HbA1c, LDL-C, hs-CRP, and TyG;
d Adjusted for sex, age, smoking, drinking, WC, SBP, HbA1c, hs-CRP, and TyG;
e Adjusted for sex, age, smoking, drinking, WC, SBP, HbA1c, LDL-C, and TyG;
f Adjusted for sex, age, smoking, drinking, WC, SBP, HbA1c, LDL-C, and hs-CRP.

Abbreviations: CMM cardiometabolic multimorbidity, BMI body mass index, WC waist circumference, HDL-C high-density lipoprotein cholesterol, hs-CRP high-sensitivity C-reactive protein, LDL-C low-density lipoprotein cholesterol, SBP systolic blood pressure, TyG triglyceride-glucose.

Table S14. Sensitivity analysis of the association between variables and incident CMM using the Fine-Gray competing risks model using the multiply imputed dataset.

Variables	Model 1		Model 2	
	SHR (95%CI)	P value	SHR (95%CI)	P value
Age				
≤60	1.00 (Reference)	-	1.00 (Reference)	-
>60	1.45 (1.18-1.78)	<0.001	1.22 (0.99-1.82) [a]	0.064
BMI				
≤ 25.6	1.00 (Reference)	-	1.00 (Reference)	-
> 25.6	2.22 (1.80-2.73)	<0.001	1.90 (1.53-2.37) [b]	<0.001
WC				
≤ 90.6	1.00 (Reference)	-	1.00 (Reference)	-
> 90.6	2.24 (1.82-2.75)	<0.001	1.84 (1.48-2.28) [c]	<0.001
LDL-C				
≤ 154.3	1.00 (Reference)	-	1.00 (Reference)	-
> 154.3	1.63 (1.27-2.11)	<0.001	1.39 (1.08-1.80) [d]	0.011
CRP				
≤ 0.86	1.00 (Reference)	-	1.00 (Reference)	-
> 0.86	1.65 (1.34-2.05)	<0.001	1.39 (1.12-1.73) [e]	0.003
TyG				
≤ 8.7	1.00 (Reference)	-	1.00 (Reference)	-
> 8.7	1.62 (1.32-1.98)	<0.001	1.29 (1.05-1.59) [f]	0.018

SHR, subdistribution hazard ratio; **CI,** confidence interval. All models were estimated using the Fine-Gray competing risks model with death treated as the competing event.

Model 1: Unadjusted for any covariates.

Model 2: Adjusted for sex, smoking, drinking, SBP, HbA1c, and the listed metabolic variables (age, WC, LDL-C, hs-CRP, TyG) excluding the exposure variable in each panel.

a Adjusted for sex, smoking, drinking, WC, SBP, HbA1c, LDL-C, hs-CRP, and TyG;
b Adjusted for sex, age, smoking, drinking, SBP, HbA1c, LDL-C, hs-CRP, and TyG;
c Adjusted for sex, age, smoking, drinking, SBP, HbA1c, LDL-C, hs-CRP, and TyG;
d Adjusted for sex, age, smoking, drinking, WC, SBP, HbA1c, hs-CRP, and TyG;
e Adjusted for sex, age, smoking, drinking, WC, SBP, HbA1c, LDL-C, and TyG;
f Adjusted for sex, age, smoking, drinking, WC, SBP, HbA1c, LDL-C, and hs-CRP.

Abbreviations: CMM cardiometabolic multimorbidity, BMI body mass index, WC waist circumference, HDL-C high-density lipoprotein cholesterol, hs-CRP high-sensitivity C-reactive protein, LDL-C low-density lipoprotein cholesterol, SBP systolic blood pressure, TyG triglyceride-glucose.

Table S15. Assessment of the proportional hazard's assumption using scaled Schoenfeld residuals across six Cox models

Variables	Age group		BMI group		WC group		LDL-C group		Hs-CRP group		TyG group	
	χ^2	P value	χ^2	P value	χ^2	P value	χ^2	P value	χ^2	P value	χ^2	P value
Age	-	-	2.087	0.149	2.035	0.154	1.932	0.165	1.966	0.161	1.962	0.161
Sex	1.869	0.172	1.805	0.179	1.798	0.180	1.781	0.812	1.711	0.191	1.907	0.167
Smoking	0.710	0.701	0.720	0.698	0.709	0.701	0.687	0.709	0.649	0.723	0.759	0.684
Drinking	6.276	0.043	6.049	0.049	6.310	0.043	6.195	0.045	6.114	0.047	6.379	0.041
SBP	1.083	0.289	1.349	0.245	1.187	0.276	1.193	0.275	1.148	0.284	1.165	0.280
WC	0.730	0.393	-	-	-	-	0.698	0.404	0.621	0.431	0.660	0.417
HbA1c	4.290	0.038	4.247	0.039	4.350	0.037	4.286	0.038	4.458	0.035	4.353	0.037
LDL-C	12.151	<0.001	12.458	<0.001	12.172	<0.001	-	-	12.221	<0.001	12.348	<0.001
Hs-CRP	0.477	0.490	0.419	0.518	0.448	0.503	0.483	0.487	-	-	0.440	0.507
TyG	3.146	0.076	3.232	0.072	3.017	0.082	3.039	0.081	3.061	0.080	-	-
Age group	0.051	0.822	-	-	-	-	-	-	-	-	-	-
LDL-C group	-	-	-	-	-	-	5.877	0.015	-	-	-	-
WC group	-	-	-	-	0.982	0.322	-	-	-	-	-	-
BMI group	-	-	0.002	0.963	-	-	-	-	-	-	-	-
Hs-CRP group	-	-	-	-	-	-	-	-	0.250	0.617	-	-
TyG group	-	-	-	-	-	-	-	-	-	-	2.790	0.095

The proportional hazards (PH) assumption was assessed using scaled Schoenfeld residuals for each of the six Cox proportional hazards models with alternative exposure variables. χ^2, chi-squared statistic. "-" indicates that the variable was not included as a covariate in the corresponding model (e.g., when serving as the primary exposure). Bold p-values denote statistically significant violations of the PH assumption ($p < 0.05$).

Abbreviations: SBP, systolic blood pressure; WC, waist circumference; HbA1c, glycated hemoglobin; LDL-C, low-density lipoprotein cholesterol; Hs-CRP, high-sensitivity C-reactive protein; TyG, triglyceride-glucose index; BMI, body mass index.

Table S16. Global tests in multivariable-adjusted Cox models stratified by LDL-C and HbA1c across six primary exposures models

Primary Exposure	Global χ^2	df	p value
Age group	11.74	10	0.303
BMI group	12.90	10	0.229
WC group	13.22	10	0.212
LDL-C group	18.23	11	0.076
CRP group	12.48	10	0.254
TyG group	12.92	10	0.228

The proportional hazards (PH) assumption was assessed in multivariable-adjusted Cox models stratified by clinical cut points of LDL-C (<130 vs. ≥130 mg/dL) and HbA1c (<6.5% vs. ≥6.5%), with adjustment for sex, smoking, drinking, and the listed metabolic variables (age, WC, SBP, hs-CRP, TyG), excluding the primary exposure variable in each panel to avoid over-adjustment. For the LDL-C group model, stratification was applied only to HbA1c, as LDL-C served as the primary exposure. The global PH test was performed using scaled Schoenfeld residuals; all p values > 0.05, confirming that the PH assumption was satisfied in all stratified models. Models were adjusted for sex, smoking, drinking, SBP, HbA1c, and the listed metabolic variables (age, WC, LDL-C, hs-CRP, TyG), excluding the exposure variable in each pane

Abbreviations: LDL-C, low-density lipoprotein cholesterol; HbA1c, glycated hemoglobin; BMI, body mass index; WC, waist circumference; SBP, systolic blood pressure; hs-CRP, high-sensitivity C-reactive protein; TyG, triglyceride-glucose index; PH, proportional hazards.

Table S17. Associations of metabolic indicators with incident CMM: Cox proportional hazards models stratified by LDL-C and HbA1c following Schoenfeld residuals testing

Variables	Model 1		Model 2	
	HR (95%CI)	P value	HR (95%CI)	P value
Age				
≤60	1.00 (Reference)	-	1.00 (Reference)	-
>60	1.70 (1.30-2.23)	<0.001	1.41 (1.06-1.88)	0.017
BMI				
≤ 25.6	1.00 (Reference)	-	1.00 (Reference)	-
> 25.6	2.37 (1.81-3.11)	<0.001	2.06 (1.53-2.77)	<0.001
WC				
≤ 90.6	1.00 (Reference)	-	1.00 (Reference)	-
> 90.6	2.44 (1.86-3.19)	<0.001	1.93 (1.44-2.57)	<0.001
LDL-C				
≤ 154.3	1.00 (Reference)	-	1.00 (Reference)	-
> 154.3	1.91 (1.37-2.67)	<0.001	1.59 (1.14-2.23)	0.007
CRP				
≤ 0.86	1.00 (Reference)	-	1.00 (Reference)	-
> 0.86	2.01 (1.50-2.68)	<0.001	1.54 (1.14-2.08)	0.005
TyG				
≤ 8.7	1.00 (Reference)	-	1.00 (Reference)	-
> 8.7	1.90 (1.45-2.49)	<0.001	1.43 (1.08-1.90)	0.012

Model 1: unadjusted.

Model 2: adjusted Cox model stratified by LDL-C (<130 vs. ≥130 mg/dL) and HbA1c (<6.5% vs. ≥6.5%), with adjustment for sex, smoking, drinking, and the listed metabolic variables (age, WC, SBP, hs-CRP, TyG), excluding the primary exposure variable in each panel to avoid over-adjustment.

Abbreviations: HR, hazard ratio; CI, confidence interval; BMI, body mass index; WC, waist circumference; LDL-C, low-density lipoprotein cholesterol; CRP, C-reactive protein; TyG, triglyceride-glucose index.
